# Transcriptional Regulation of the Peripheral Pathway for the Anaerobic Catabolism of Toluene and *m*-Xylene in *Azoarcus* sp. CIB

**DOI:** 10.3389/fmicb.2018.00506

**Published:** 2018-03-22

**Authors:** Blas Blázquez, Manuel Carmona, Eduardo Díaz

**Affiliations:** Department of Microbial and Plant Biotechnology, Centro de Investigaciones Biológicas–Consejo Superior de Investigaciones Científicas, Madrid, Spain

**Keywords:** toluene, *m*-xylene, *Azoarcus*, anaerobic degradation, *tdiSR*, catabolite repression, biomarker

## Abstract

Alkylbenzenes, such as toluene and *m*-xylene, are an important class of contaminant hydrocarbons that are widespread and tend to accumulate in subsurface anoxic environments. The peripheral pathway for the anaerobic oxidation of toluene in bacteria consists of an initial activation catalyzed by a benzylsuccinate synthase (encoded by *bss* genes), and a subsequent modified β-oxidation of benzylsuccinate to benzoyl-CoA and succinyl-CoA (encoded by *bbs* genes). We have shown here that the *bss* and *bbs* genes, which are located within an integrative and conjugative element, are essential for anaerobic degradation of toluene but also for *m*-xylene oxidation in the denitrifying beta-proteobacterium *Azoarcus* sp. CIB. New insights into the transcriptional organization and regulation of a complete gene cluster for anaerobic catabolism of toluene/*m*-xylene in a single bacterial strain are presented. The *bss* and *bbs* genes are transcriptionally coupled into two large convergent catabolic operons driven by the *PbssD* and *PbbsA* promoters, respectively, whose expression is inducible when cells grow anaerobically in toluene or *m*-xylene. An adjacent *tdiSR* operon driven by the *PtdiS* promoter encodes a putative two-component regulatory system. TdiR behaves as a transcriptional activator of the *PbssD*, *PbbsA*, and *PtdiS* promoters, being benzylsuccinate/(3-methyl)benzylsuccinate, rather than toluene/*m*-xylene, the inducers that may trigger the TdiS-mediated activation of TdiR. In addition to the TdiSR-based specific control, the expression of the *bss* and *bbs* genes in *Azoarcus* sp. CIB is under an overimposed regulation that depends on certain environmental factors, such as the presence/absence of oxygen or the availability of preferred carbon sources (catabolite repression). This work paves the way for future strategies toward the reliable assessment of microbial activity in toluene/*m*-xylene contaminated environments.

## Introduction

Aromatic compounds such as alkylbenzenes, e.g., toluene, xylenes, ethylbenzene and benzene, are an important class of contaminants that are prominently placed amongst the US Agency for Toxic Substances and Disease Registry’s list of priority pollutants because of their carcinogenic and/or neurotoxic effects to humans. Since alkylbenzenes are relatively soluble and mobile in water, they are widespread and tend to accumulate in hostile environments, e.g., subsurface anoxic environments, where they are of major concern to groundwater quality and ecosystem health. Therefore, the anaerobic degradation of alkylbenzenes is an important practical aspect of bioremediation and becomes crucial for the biogeochemical cycles (Kummel et al., 2013; Rabus et al., 2016; Lueders, 2017). Despite the aerobic degradation of alkylbenzenes has been extensively studied, the microbial catabolism of these aromatic hydrocarbons under anaerobic conditions is much less known, and novel pathways and enzymes have been recently described, some of which are of potential biotechnological interest (Rabus et al., 2016; Lueders, 2017). Toluene has been used widely as a model compound for studying anaerobic alkylbenzene degradation. Toluene can be degraded coupled to anaerobic respiration with nitrate, sulfate, iron (III), manganese (IV) or carbonate serving as terminal electron acceptors (Foght, 2008; Carmona et al., 2009; Rabus et al., 2016). *Geobacter metallireducens* GS-15 was the first pure bacterial culture described to be able to degrade toluene in anaerobic conditions (Lovley et al., 1989). Several isolates capable of anaerobic toluene degradation have been described since then, including both facultative anaerobes, e.g., beta-proteobacteria of the *Azoarcus*, “*Aromatoleum*,” *Thauera, Georgfuchsia, Herminiimonas* genera, and alpha-proteobacteria of the *Magnetospirillum* genus, and obligate anaerobes, e.g., delta-proteobacteria of the *Geobacter, Desulfobacula* and *Desulfobacterium* genera, and some clostridial strains (Wöhlbrand et al., 2013; Bozinovski et al., 2014; Kim et al., 2014; Strijkstra et al., 2014; Rabus et al., 2016; Lueders, 2017; Meyer-Cifuentes et al., 2017). Most of the studies on anaerobic toluene degradation have been made on the denitrifying bacteria *Thauera aromatica* K172 and T1 strains, *Azoarcus* sp. strain T and “*Aromatoleum aromaticum*” EbN1 strain (Coschigano and Young, 1997; Leuthner et al., 1998; Krieger et al., 1999; Achong et al., 2001; Hermuth et al., 2002; Leutwein and Heider, 2002; Chakraborty and Coates, 2004; Coschigano and Bishop, 2004; Kube et al., 2004; Kühner et al., 2005).

**FIGURE 1:**
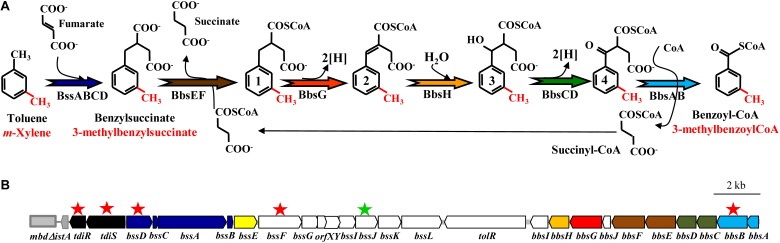
Proposed toluene and *m*-xylene anaerobic peripheral degradation pathway in *Azoarcus* sp. CIB: **(A)** Scheme of the proposed peripheral pathway of anaerobic degradation of toluene and *m*-xylene. The enzymes are indicated following the color code indicated in **(B)**. Toluene and *m*-xylene derivatives are shown in black and red color, respectively. Intermediates 1 correspond to benzylsuccinyl-CoA (black) and (3-methyl)benzylsuccinyl-CoA (red). Intermediates 2 correspond to phenylitaconyl-CoA (black) and (3-methyl)phenylitaconyl-CoA (red). Intermediates 3 correspond to 2-[hydroxyphenyl]-succinyl-CoA (black) and 2-[hydroxyphenyl(methyl)]-succinyl-CoA (red). Intermediates 4 correspond to benzoylsuccinyl-CoA (black) and (3-methyl)benzoylsuccinyl-CoA (red). Modified from Carmona et al. (2009). **(B)** Scheme of the gene cluster encoding the anaerobic peripheral pathway of toluene and *m*-xylene in *Azoarcus* sp. CIB. Genes are represented by arrows and their predicted function is annotated as follows: black, regulatory genes; dark blue, genes encoding the (3-methyl)benzylsuccinate synthase (BSS); yellow, gene encoding a putative BSS chaperone; orange, gene encoding the (3-methyl)phenylitaconyl-CoA hydratase; red, gene encoding the (3-methyl)benzylsuccinyl-CoA dehydrogenase; brown, genes encoding the succinyl-CoA:(3-methyl)benzylsuccinate CoA transferase; green, genes encoding a 2-[hydroxyphenyl(methyl)]-succinyl-CoA dehydrogenase; light blue, genes encoding the (3-methyl)benzoylsuccinyl-CoA thiolase; white, genes of unknown function. A truncated IS*21* transposase *istA* gene (Δ*istA*) is shown by a gray arrow. The gray rectangle indicates the *mbd* cluster responsible for the 3-methylbenzoyl-CoA central pathway. Genes that have been inactivated in this work and avoid the use of toluene and *m*-xylene as sole carbon and energy source by the corresponding mutant strains (*Azoarcus* sp. CIBd*tidR*, *Azoarcus* sp. CIBd*tidS, Azoarcus* sp. CIBd*bssD, Azoarcus* sp. CIBd*bssF, Azoarcus* sp. CIBd*bbsB)* are indicated with a red star. The gene that has been inactivated and does not avoid the use of toluene and *m*-xylene as sole carbon and energy source in the corresponding mutant strain (*Azoarcus* sp. CIBd*bssJ)* is indicated with a green star.

**FIGURE 2:**
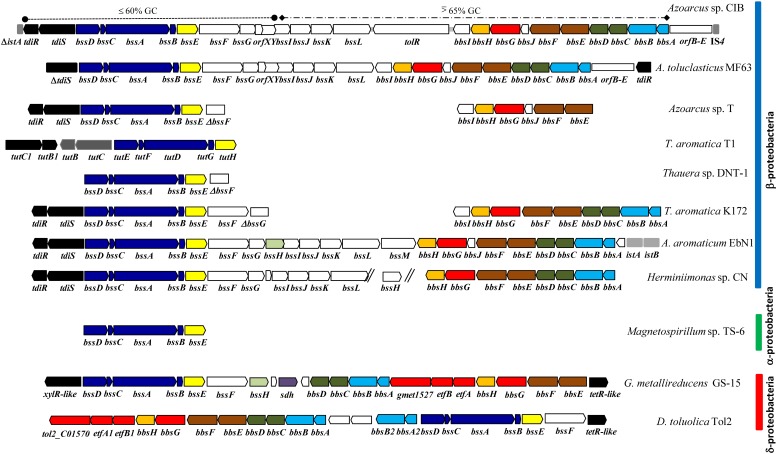
Scheme of the genetic organization of the *tdi-bss-bbs* cluster in different bacteria. The *tdi-bss-bss* clusters from *Azoarcus* sp. CIB, *Azoarcus toluclasticus* MF63 (Acc. No. NZ_ARJX00000000.1), *Azoarcus* sp. strain T (Ac. No. AY032676), *T. aromatica* T1 (Ac. No. U57900 and AF113168), *Thauera* sp. strain DNT-1 (Ac. No. AB066263), *T. aromatica* K172 (Ac. No. AJ001848 and AF173961), “*Aromatoleum aromaticum*” EbN1 (Ac. No. NC_006513), *Herminiimonas* sp. CN (Ac. No. AVCC01000000), *Magnetospirillum* sp. strain TS-6 (Ac. No. AB167725), *Geobacter metallireducens* GS-15 (Ac. No. NC_007517) and *Desulfobacula toluolica* Tol2 (Ac. No. NC_018645) are represented here. Genes are represented by arrows following the color code indicated in **Figure 1**: black, regulatory genes; gray, putative regulatory genes of an aerobic toluene degradation pathway; dark blue, genes encoding the (3-methyl)benzylsuccinate synthase (BSS); yellow, genes encoding a putative BSS chaperone; orange, genes encoding the (3-methyl)phenylitaconyl-CoA hydratase; red, genes encoding the (3-methyl)benzylsuccinyl-CoA dehydrogenase and its predicted associated electron-transfer system; brown, genes encoding the succinyl-CoA:(3-methyl)benzylsuccinate CoA transferase; green, genes encoding a 2-[hydroxyphenyl(methyl)]-succinyl-CoA dehydrogenase; light blue, genes encoding the (3-methyl)benzoylsuccinyl-CoA thiolase; light green, genes encoding a putative toluene transport system; violet, gene encoding a putative succinate-dehydrogenase flavoprotein; white, genes of unknown function. Insertion sequences are indicated by gray rectangles. The white rectangle next to the *bbs* operon in *Azoarcus* sp. CIB represents *orfB* (AzCIB_4527), *orfC* (AzCIB_4528), *orfD* (AzCIB_4529) and *orfE* (AzCIB_4530) genes, that are also present in *A. toluclasticus* MF63. In the *bss-bbs* cluster of *Azoarcus* sp. CIB, the DNA fragment harboring the *bssDCABEFG* genes and whose average GC content is 58% is indicated by a disontinuous line flanked by circles. The DNA fragment containing the rest of the *bss* (*bssIJKL*) and *bbs* genes, whose average GC content is 65%, is indicated by a discontinuous line flanked by diamonds.

The first step in the anaerobic catabolism of toluene is the addition of the methyl group of toluene to fumarate to form (*R*)-benzylsuccinate by the strictly anoxic benzylsuccinate synthase (BSS), a glycyl radical enzyme (**Figure 1A**). The genes *bssA*, *bssB* and *bssC* code for the α (BssA), β (BssB) and γ (BssC) subunits of the heterohexameric (αβγ)_2_ BSS (Leuthner et al., 1998; Krieger et al., 2001; Bhandare et al., 2006; Funk et al., 2014; Heider et al., 2016). Conversion to the active, radical-containing form of BSS depends on an activating enzyme (BssD) that belongs to the family of *S*-adenosyl-methionine radical enzymes and that is encoded by a gene (*bssD*) which is closely associated with the *bssABC* genes constituting the *bssDCAB* operon of toluene-degrading bacteria (**Figure 2**) (Selmer et al., 2005; Heider et al., 2016). The *bss* operons contain two additional conserved genes, *bssE* and *bssF* (**Figure 2**), whose functions are still unknown. Whereas BssE is an essential protein for toluene degradation (Bhandare et al., 2006), the role of BssF in the anaerobic oxidation of toluene has not yet been explored. Moreover, in beta-proteobacteria the *bss* cluster contains a set of additional genes, i.e., the *bssGIJKL* genes (**Figure 2**), whose function is so far unknown. Despite the primary structure of the gene products is conserved in the *bss* clusters from different denitrifying bacteria, the transcriptional organization of the *bss* clusters differs between species and even between strains of the same species (Coschigano, 2000; Achong et al., 2001; Kube et al., 2004).

Further anaerobic degradation of toluene consists of a modified beta-oxidation of (*R*)-benzylsuccinate to benzoyl-CoA and succinyl-CoA (**Figure 1A**). All enzymes of this pathway are encoded by the *bbs* gene cluster (**Figure 2**) (Leuthner and Heider, 2000; Leutwein and Heider, 2001, 2002; Kube et al., 2004). Like in the *bss* gene cluster, the *bbs* cluster contains genes, e.g., *bbsI* and *bbsJ*, of unknown function (Carmona et al., 2009). Although the gene organization of the *bbsABCDEFJGH* cluster is usually conserved except in some delta-proteobacteria (**Figure 2**) (Kube et al., 2004; Butler et al., 2007; Chaurasia et al., 2015), the transcriptional organization of a *bbs* cluster has not been studied so far in any bacteria.

Upstream of the *bss* cluster in most denitrifying bacteria there are two genes, *tdiS* and *tdiR* (toluene-degradation inducer) (**Figure 2**) that were proposed to encode the sensor histidine kinase and the cognate response regulator, respectively, of a two-component regulatory system likely involved in the transcriptional control of the catabolic *bss* and *bbs* genes (Coschigano, 2000; Achong et al., 2001; Hermuth et al., 2002; Kube et al., 2004; Kühner et al., 2005). Extracts of *Escherichia coli* cells that expressed the *tdiR* gene from *T. aromatica* K172 were able to retard the migration of a DNA probe that contained the *bssD* promoter, supporting the notion that TdiR likely behaves as a transcriptional regulator of the *bss* genes (Leuthner and Heider, 1998). Nevertheless, a clear experimental demonstration that TdiS and TdiR control the expression of both *bss* and *bbs* genes in beta-proteobacteria is still missing.

Unlike toluene degradation, anaerobic degradation of xylenes (*meta-*, *ortho*-, and *para*-xylene) has been much less studied. The first reaction of the *m*-xylene degradation pathway is performed by the BSS enzyme that catalyzes the addition of fumarate to the methyl group to form (3-methyl)benzylsuccinate (**Figure 1A**) (Krieger et al., 1999; Verfürth et al., 2004). The (3-methyl)benzylsuccinate was also detected as a key intermediate of the fumarate addition pathway in xylene-degrading sulfate-reducing cultures (Morasch et al., 2004; Herrmann et al., 2009). On the other hand, it has been proposed that the *bbs* gene products might be responsible of the β-oxidation of (3-methyl)benzylsuccinate to 3-methylbenzoyl-CoA and succinyl-CoA in a similar way than in the catabolism of toluene (**Figure 1A**) (Krieger et al., 1999; Bozinovski et al., 2014), although there is not yet a genetic demonstration that *bbs* genes are responsible for anaerobic *m*-xylene degradation.

*Azoarcus* sp. CIB is facultative anaerobic bacterium able to grow aerobically in toluene and anaerobically (nitrate-reducing) in toluene and *m*-xylene (López-Barragán et al., 2004). Moreover, *Azoarcus* sp. CIB also shows an endophitic lifestyle (Fernández et al., 2014) and is able to resist some metals and metalloids (Fernández-Llamosas et al., 2016). The strain CIB has been used previously to decipher the transcriptional organization and regulation of several gene clusters involved in the anaerobic degradation of aromatic compounds and in the resistance strategies to survive in the presence of high concentrations of these contaminants (López-Barragán et al., 2004; Blázquez et al., 2008; Carmona et al., 2009; Valderrama et al., 2012, 2014; Juárez et al., 2013; Juárez et al., 2015; Martín-Moldes et al., 2016; Zamarro et al., 2016). An *in silico* analysis of the *Azoarcus* sp. CIB genome sequence revealed the presence of a chromosomal region within the integrative and conjugative ICE*_XTD_* element coding for proteins with significant amino acid sequence identity with Bss and Bbs proteins in closely related bacteria from the *Azoarcus*, “*Aromatoleum*” and *Thauera* genera (Martín-Moldes et al., 2015; Zamarro et al., 2016). This sequence comparison analysis allowed us to propose a similar biochemical pathway for the anaerobic conversion of toluene and *m*-xylene to benzoyl-CoA and 3-methylbenzoyl-CoA, respectively, in strain CIB (**Figure 1**). Further degradation of benzoyl-CoA and 3-methylbenzoyl-CoA in *Azoarcus* sp. CIB proceeds via the central bzd (stands for benzoate degradation) and mbd (stands for methylbenzoate degradation) pathways, respectively (López-Barragán et al., 2004; Juárez et al., 2013). In this work we have used *Azoarcus* sp. CIB to study for the first time the transcriptional organization and regulation of the complete *bss-bbs* cluster in a facultative anaerobe. Our results reveal that both the *bss* and *bbs* genes are essential for the anaerobic degradation of toluene and *m*-xylene.

## Materials and Methods

### Bacterial Strains, Plasmids and Growth Conditions

The *E. coli* and *Azoarcus* strains, as well as the plasmids used in this study, are detailed in **Table 1**. *E. coli* strains were grown at 37°C in Lysogeny Broth (LB) mediun (Bertani, 1951). *Azoarcus* sp. strain CIB and its derivatives were grown anaerobically under nitrate reducing conditions (10 mM nitrate) at 30°C in MC medium as previously described (López-Barragán et al., 2004). Aromatic hydrocarbons such as toluene, xylenes, styrene were supplied at 250 mM in an inert carrier phase of 2,2,4,4,6,8,8-heptamethylnonan (HMN). Benzylsuccinate was added at 4 mM to the culture medium as inducer. Organic acids such as succinate or pyruvate were added at 0.2% (w/v). *Azoarcus* sp. CIB cells were also cultivated aerobically in MC medium in the absence of nitrate. When using toluene aerobically as sole carbon source, it was added directly to the culture medium at 1 mM. Where appropriate, antibiotics were added to the culture medium at the following concentrations: ampicillin, 100 μg/ml; gentamicin, 10 μg/ml; kanamycin, 50 μg/ml. Growth was determined by measuring the optical density at 600 nm (OD_600_) in a Shimadzu UV-260 spectrophotometer.

**Table 1:** Bacterial strains and plasmids used in this work.

Strains and plasmids	Description^a^	Reference or source
Strains		
*E. coli*		
DH10B	F’, *mcrA*, D(*mrr*, *hsdRMS-mcrBC*), f80d*lac*DM15, D*lacX74*, *deoR*, *recA1*, *araD139*, D(*ara-leu*)*7697*, *galU*, *galK* l, *rpsL*, *endA1*, *nupG*	Life Technologies
S17-1λpir	*recA, thi, hsdRM*+, RP4::2-Tc::Mu::Km, Tn*7*, λpir phage lysogen; Tp^r^, Sm^r^	de Lorenzo and Timmis, 1994
*Azoarcus* sp. CIB	Wild-type strain; T^+^ X^+^	López-Barragán et al., 2004
*Azoarcus* sp. CIBd*tdiR*	CIB mutant strain with a disruption of the *tdiR* gene; Km^r^, T^-^ X^-^	This study
*Azoarcus* sp. CIBd*tdiS*	CIB mutant strain with a disruption of the *tdiS* gene; Km^r^, T^-^ X^-^	This study
*Azoarcus* sp. CIBd*bssD*	CIB mutant strain with a disruption of the *bssD* gene; Km^r^, T^-^ X^-^	This study
*Azoarcus* sp. CIBd*bssF*	CIB mutant strain with a disruption of the *bssF* gene; Km^r^, T^-^ X^-^	This study
*Azoarcus* sp. CIBd*bssJ*	CIB mutant strain with a disruption of the *bssJ* gene; Km^r^, T^+^ X^+^	This study
*Azoarcus* sp. CIBd*bbsB*	CIB mutant strain with a disruption of the *bbsB* gene; Km^r^, T^-^ X^-^	This study
Plasmids		
pGEM-T Easy	Ap^r^, *ori*ColE1, *lacZ*α, used for cloning PCR products	Promega
pGEM-tdiR	Ap^r^, pGEM-T Easy containing a 400 bp XbaI/PstI *tdiR* internal fragment	This study
pGEM-tdiS	Ap^r^, pGEM-T Easy containing a 623 bp EcoRI *tdiS* internal fragment	This study
pGEM-bssD	Ap^r^, pGEM-T Easy containing a 593 bp PstI/BamHI *bssD* internal fragment	This study
pGEM-bssF	Ap^r^, pGEM-T Easy containing a 458 bp HindIII/XbaI *bssF* internal fragment	This study
pGEM-bssJ	Ap^r^, pGEM-T Easy containing a 353 bp PstI/SalI *bssJ* internal fragment	This study
pGEM-bbsB	Ap^r^, pGEM-T Easy containing a 512 bp PstI/BamHI *bbsB* internal fragment	This study
pK18*mob*	Km^r^, *ori*ColE1, Mob^+^, *lacZ*α, used for directed insertional disruption	Schäfer et al., 1994
pK18*mobtdiR*	Km^r^, pK18*mob* containing a 400 bp XbaI/PstI *tdiR* internal fragment	This study
pK18*mobtdiS*	Kmr, pK18*mob* containing a 623 bp EcoRI *tdiS* internal fragment	This study
pK18*mobbssD*	Km^r^, pK18*mob* containing a 593 bp PstI/BamHI *bssD* internal fragment	This study
pK18*mobbssF*	Km^r^, pK18*mob* containing a 458 bp HindIII/XbaI *bssF* internal fragment	This study
pK18*mobbssJ*	Km^r^, pK18*mob* containing a 353 bp PstI/SalI *bssJ* internal fragment	This study
pK18*mobbbsB*	Km^r^, pK18*mob* containing a 512 bp PstI/BamHI *bbsB* internal fragment	This study
pSJ3	Ap^r^, *ori*ColE1, ’*lacZ* promoter probe vector	Ferrández et al., 1998
pSJPbbsA	Ap^r^, pSJ3 derivative carrying the *PbbsA*:*:lacZ* fusion	This study
pSJPtdiS	Ap^r^, pSJ3 derivative carrying the *PtdiS*:*:lacZ* fusion	This study
pBBR5T	Gm^r^, pBBR1MCS-5 derivative with a T7 transcriptional terminator upstream of the ’*lacZ* gene	Blázquez et al., 2008
pBBRPbbsA	Gm^r^, pBBR5T derivative carrying the *PbbsA*:*:lacZ* fusion	This study
pBBRPbssD	Gm^r^, pBBR5T derivative carrying the *PbssD*:*:lacZ* fusion	This study
pBBRPtdiS	Gm^r^, pBBR5T derivative carrying the *PtdiS*:*:lacZ* fusion	This study


*^*a*^The abbreviations used are: T^+^, anaerobic growth in toluene; X^+^, anaerobic growth in m-xylene; T^-^, lack of anaerobic growth in toluene; X^-^, lack of anaerobic growth in m-xylene; Ap^*r*^, ampicillin-resistant; Gm^*r*^, gentamicin-resistant; Km^*r*^, kanamycin-resistant; Sm^*r*^, streptomycin-resistant.*

### Molecular Biology Techniques

Standard molecular biology techniques were performed as previously described (Sambrook and Russell, 2001). DNA fragments were purified with Gene-Clean Turbo (BIO101 Systems); plasmids and PCR products were purified with a High Pure Plasmid and PCR Product Purifications kits (Roche), respectively. Oligonucleotides were supplied by Sigma Co. All cloned inserts and DNA fragments were confirmed by DNA sequencing with fluorescently labeled dideoxynucleotide terminators (Sanger et al., 1977) and AmpliTaq FS DNA polymerase (Applied Biosystems) in an ABI Prism 377 automated DNA sequencer (Applied Biosystems). Transformation of *E. coli* cells was carried out by using the RbCl method or by electroporation (Gene Pulser, Bio-Rad) (Sambrook and Russell, 2001). Plasmids were transferred from *E. coli* S17-1λpir (donor strain) into *Azoarcus* sp. recipient strains by biparental filter mating as described previously (López-Barragán et al., 2004).

### Construction of *Azoarcus* sp. CIBd*tdiR*, *Azoarcus* sp. CIBd*tdiS*, *Azoarcus* sp. CIBd*bssD*, *Azoarcus* sp. CIBd*bssF*, *Azoarcus* sp. CIBd*bssJ*, and *Azoarcus* sp. CIBd*bbsB* Strains

For insertional disruption of the genes through single homologous recombination, an internal region of each gene was PCR-amplified, cloned into plasmid pGEM-T Easy, and subcloned into the pK18*mob* suicide vector (**Table 1**). For gene disruption of the *tdiR* gene, a 0.4-kb internal fragment was PCR-amplified using primers tdiRint5 and tdiR131.5 (Supplementary Table S1), and cloned in plasmid pGEM-tdiR (**Table 1**). The pGEM-tdiR plasmid was XbaI/PstI double-digested and the 0.4-kb fragment was then subcloned giving rise to the suicide plasmid pK18*mobtdiR* (**Table 1**). For gene disruption of the *tdiS* gene, a 0.6-kb internal fragment was PCR amplified using primers 5TdiS and 3TdiS (Supplementary Table S1), and cloned in plasmid pGEM-tdiS (**Table 1**). The pGEM-tdiS plasmid was EcoRI digested and the 623-bp fragment was then subcloned producing plasmid pK18*mobtdiS* (**Table 1**). The *tdiS* mutant allows expression of the downstream *tdiR* gene by transcription from the promoter of the kanamycin resistance gene present in the suicide plasmid. For gene disruption of the *bssD* gene, a 0.6-kb internal fragment was PCR amplified using primers 5BssD and 3BssD (Supplementary Table S1), and cloned in plasmid pGEM-bssD (**Table 1**). The pGEM-bssD plasmid was PstI/BamHI double-digested and the 593-bp fragment was then subcloned into pK18*mob* producing plasmid pK18*mobbssD* (**Table 1**). For gene disruption of the *bssF* gene, a 0.4-kb internal fragment was PCR-amplified using primers 5BssF and 3BssF (Supplementary Table S1) and cloned in plasmid pGEM-bssF (**Table 1**). The pGEM-bssF plasmid was HindIII/XbaI double-digested and the 451-bp fragment was then subcloned giving rise to the suicide plasmid pK18*mobbssF* (**Table 1**). For gene disruption of the *bssJ* gene, a 0.3-kb internal fragment was PCR amplified using primers 5BssJ and 3BssJ (Supplementary Table S1) and cloned in plasmid pGEM-bssJ (**Table 1**). The pGEM-bssJ plasmid was PstI/SalI double-digested and the 347-bp fragment was then subcloned producing plasmid pK18*mobbssJ* (**Table 1**). For gene disruption of the *bbsB* gene, a 0.5-kb internal fragment was PCR amplified using primers 5BbsB and 3BbsB, and cloned in plasmid pGEM-bbsB (**Table 1**). The pGEM-bbsB plasmid was *Pst*I/*Bam*HI double-digested and the 512-bp fragment was then subcloned producing plasmid pK18*mobbbsB* (**Table 1**). The derivative pK18*mob* plasmids were then transferred from *E. coli* S17-1λpir (donor strain) into *Azoarcus* sp. CIB (recipient strain) by biparental filter mating as previously described (de Lorenzo and Timmis, 1994; López-Barragán et al., 2004). Exconjugant *Azoarcus* sp. CIB mutant strains harboring the disrupted genes by insertion of the corresponding suicide plasmids, were isolated aerobically on kanamycin-containing MC medium lacking nitrate and containing 0.2% citrate as the sole carbon source for counter-selection of donor cells. The mutant strains were analyzed by PCR to confirm the disruption of the target genes.

### Construction of *lacZ* Translational Fusions

To construct plasmid pSJPbbsA that harbors the *PbbsA::lacZ* translational fusion, a 356-bp *Xba*I/*Pst*I DNA fragment containing the *bbsA* (AzCIB_4526)*-orfB* (AzCIB_4527) intergenic region was PCR-amplified from *Azoarcus* sp. CIB chromosomal DNA by using oligonucleotides 5PbbsA and 3PbbsA (5′-AACTGCAGGACATGACGCCTCCGCAGCATTTG-3′ (Supplementary Table S1), and then cloned into the XbaI/PstI double-digested pSJ3 plasmid (**Table 1**). Plasmid pBBRPbbsA was constructed by subcloning a 3.5-kb EcoRI/HindIII fragment harboring the *PbbsA::lacZ* translational fusion from pSJPbbsA into the EcoRI/HindIII double-digested broad-host range plasmid pBBR5T (**Table 1**). To construct plasmid pBBRPbssD that carries the *PbssD::lacZ* fusion, a 335-bp SpeI/BamHI DNA fragment containing the *tdiS-bssD* intergenic region was PCR-amplified from *Azoarcus* sp. CIB chromosomal DNA by using oligonucleotides 5PtdiS and 3PbssD (Supplementary Table S1), and then cloned into the SpeI/BamHI double-digested pBBRPbbsA (**Table 1**). To construct plasmid pSJ3PtdiS that carries the *PtdiS::lacZ* translational fusion, a 314-bp BamHI fragment containing the *bssD-tdiS* intergenic region was PCR-amplified from *Azoarcus* sp. CIB chromosomal DNA by using oligonucleotides 3PbssD and PtdiS3’(Supplementary Table S1), and then cloned into the BamHI digested pSJ3 plasmid (**Table 1**). Plasmid pBBRPtdiS was constructed by subcloning a 3.5-kb EcoRI/HindIII DNA fragment harboring the *PtdiS::lacZ* fusion from pSJ3PtdiS into the EcoRI/HindIII double-digested broad-host range plasmid pBBR5T (**Table 1**).

### RNA Extraction and Reverse Transcription-PCR Experiments

*Azoarcus* sp. CIB cells grown in MC medium containing the appropriate carbon sources were harvested at the mid-exponential phase of growth and stored at -80°C. *Azoarcus* sp. CIBd*bbsB* and CIBd*bssD* strains grown in kanamycin-containing MC medium with pyruvate plus toluene were harvested at the stationary phase of growth and stored at -80°C. Pellets were thawed, and cells were lysed in TE buffer (10 mM Tris-HCl, pH 7.5, 1 mM EDTA) containing 50 mg ml^-1^ lysozyme. Total RNA was extracted using the RNeasy mini kit (Qiagen), including a DNase treatment according to the manufacturer instructions (Ambion), precipitated with ethanol, washed, and resuspended in RNase-free water. The concentration and purity of the RNA samples were measured by using a ND1000 Spectrophotometer (Nanodrop Technologies) according to the manufacturer’s protocols. Synthesis of total cDNA was carried out with 20 μl of reverse transcription reactions containing 400 ng of RNA, 0.5 mM concentrations of each dNTP, 200 U of SuperScript II reverse transcriptase (Invitrogen), and 5 μM concentrations of random hexamers as primers in the buffer recommended by the manufacturer. Samples were initially heated at 65°C for 5 min then incubated at 42°C for 2 h, and the reactions were terminated by incubation at 70°C for 15 min. In standard RT-PCR reactions, the cDNA was amplified with 1 U of AmpliTaq DNA polymerase (Biotools) and 0.5 μM concentrations of the corresponding primer pairs (oligonucleotides 1–20; Supplementary Table S1). Samples were initially denatured by heating at 94°C for 3 min. A 30-cycle amplification program was followed (94°C for 40 s, 60°C for 40 s and 72°C for 60 s). Control reactions in which reverse transcriptase was omitted from the reaction mixture ensured that DNA products resulted from the amplification of cDNA rather than from DNA contamination. The *dnaE* gene encoding the α-subunit of DNA polymerase III was used to provide an internal control cDNA that was amplified with oligonucleotides 5′POLIIIHK/3′POLIIIHK (Supplementary Table S1). The expression of the internal control was shown to be constant across all samples analyzed.

### β-Galactosidase Assays

The β-galactosidase activities from promoter-*lacZ* reporter fusions were measured with permeabilized cells when cultures reached mid-exponential or stationary phase of growth, as described (Miller, 1972).

### Sequence Data Analyses

Nucleotide sequence analyses were done at the National Center for Biotechnology Information (NCBI) server^1^. The amino acid sequences of the open reading frames were compared with those present in databases using the TBLASTN algorithm (Altschul et al., 1990) at the NCBI server^2^. Pairwise and multiple protein sequence alignments were made with the ClustalW program (Thompson et al., 1994) at the EMBL-EBI server^3^. Phylogenetic analysis of the different proteins was carried out according to the Kimura two-parameter method (Kimura, 1980), and a tree was reconstructed using the neighbor-joining method (Saitou and Nei, 1987) of the PHYLIP program (Felsenstein, 1993) at the TreeTop-GeneBee server^4^ and represented using TreeView X 0.5.1 (Glasgow University).

## Results and Discussion

### The *bss-bbs* Cluster Is Involved in the Anaerobic Degradation of Toluene and *m*-Xylene in *Azoarcus* sp. CIB

As indicated in the Introduction, the *in silico* analysis of the genome sequence of *Azoarcus* sp. CIB revealed a gene cluster of 26 open reading frames most of which encode orthologous of the Bss and Bbs proteins involved in the peripheral pathway for the anaerobic degradation of toluene in several bacteria. This cluster is located in the close vicinity of the *mbd* genes encoding the 3-methylbenzoyl-CoA anaerobic central pathway (Juárez et al., 2013), strongly suggesting that it constitutes the *bss-bbs* cluster for the anaerobic degradation of toluene and *m*-xylene in strain CIB (Zamarro et al., 2016) (**Figure 1B**).

**FIGURE 3:**
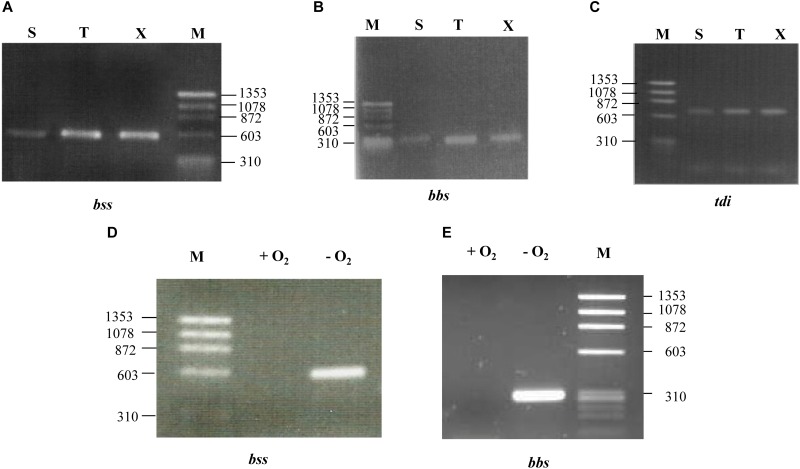
The expression of the *bss, bbs* and *tdi* genes is inducible in *Azoarcus* sp. CIB. Agarose gel electrophoresis of the RT-PCR products. Gene expression was monitored by RT-PCR as described in Section “Materials and Methods” with the primer pairs bssA5new/bssA3new, bbsA331.3/bbsA5new and tdiRint5/tdiSF.3 (Supplementary Table S1) that amplify a *bssA* gene fragment **(A,D)**, a *bbsA* gene fragment **(B,E)**, or the *tdiS-tdiR* intergenic region **(C)**, respectively. **(A–C)** *Azoarcus* sp. CIB cells were grown anaerobically until mid-exponential phase by using 0.2% succinate (S), 250 mM of toluene (T) or 250 mM *m*-xylene (X) as sole carbon source. **(D,E)** *Azoarcus* sp. CIB cells were grown under anaerobic (–O_2_) or aerobic (+O_2_) conditions using toluene as only carbon source. Lanes M, molecular size markers (HaeIII-digested ΦX174 DNA); numbers indicate the sizes of the markers (in bp).

Since the toluene peripheral pathway was shown to be inducible when bacteria grow in the presence of toluene (Coschigano, 2000; Achong et al., 2001; Hermuth et al., 2002; Kube et al., 2004; Kühner et al., 2005), we checked whether the expression of the *bss-bbs* genes was inducible when *Azoarcus* sp. CIB cells were grown anaerobically on toluene or *m*-xylene with respect to the use of succinate or benzoate as sole carbon source. The analysis of the RT-PCR amplification products revealed that the expression of the *bssA* and *bbsA* genes is induced when the cells grow in the presence of toluene or *m*-xylene as sole carbon sources (**Figures 3A,B**), suggesting their participation in the anaerobic degradation of both aromatic hydrocarbons.

Usually the transcription of anaerobic degradation pathways is regulated by the presence of oxygen in the culture medium (Durante-Rodríguez et al., 2006; Carmona et al., 2009; Juárez et al., 2013). To determine if the *bss-bbs* cluster from *Azoarcus* sp. CIB is under oxygen control, gene expression studies were performed with cells grown with toluene as sole carbon source under aerobic or anaerobic conditions. RT-PCR experiments showed that the *bssA* and *bbsA* genes are expressed only under anoxic conditions (**Figures 3D,E**). A similar oxygen-dependent expression of the *bss* genes was reported in *Magnetospirillum* sp. TS-6 (Shinoda et al., 2005), and it appears to be consistent with the fact that BSS is a strictly anaerobic enzyme (Beller and Spormann, 1998; Leuthner et al., 1998; Heider et al., 2016). Nevertheless, the oxygen-dependent regulation of the anaerobic toluene degradation pathway may differ from one strain to another since the *bss* genes from *Thauera* sp. DNT-1 were shown to be transcribed both in aerobic and anaerobic conditions (Shinoda et al., 2004).

To confirm that the *bss-bbs* cluster described above was responsible for the anaerobic degradation of toluene and *m*-xylene in *Azoarcus* sp. CIB, we constructed CIB mutant strains with insertional disruptions within some of the *bss* and *bbs* genes that are orthologous to those that have been reported or suggested to be essential for toluene degradation in other bacteria. Thus, we constructed the *Azoarcus* sp. CIBd*bssD*, and CIBd*bbsB* mutant strains harboring the *bssD* and *bbsB* disrupted genes, respectively (**Table 1**). As expected, none of the two mutant strains was able to use toluene as the sole carbon and energy source under anaerobic conditions (**Table 1**). Interestingly, none of the mutant strains was also able to use anaerobically *m*-xylene as carbon source (**Table 1**). Although inactivation of the *bssA* gene in *Azoarcus* sp. strain T was shown previously to prevent anaerobic growth on *m*-xylene (Achong et al., 2001), and (3-methyl)benzylsuccinate was proposed to be converted into 3-methylbenzoyl-CoA via a modified β-oxidation pathway (**Figure 1A**) (Krieger et al., 1999), a genetic demonstration that the *bbs* genes were indeed needed for the anaerobic growth on *m*-xylene was still lacking. In fact, some studies had suggested the existence of separate Bbs isoenzymes in toluene and *m*-xylene catabolism (Leutwein and Heider, 2002). However, our results reveal that both the *bss* and *bbs* genes are essential for the anaerobic degradation of toluene and *m*-xylene in *Azoarcus* sp. CIB.

So far, only the *bssDCABE* genes have been shown to be essential for the anaerobic conversion of toluene to benzylsuccinate (Leuthner et al., 1998; Krieger et al., 2001; Bhandare et al., 2006; Funk et al., 2014; Heider et al., 2016). However, the *bss* cluster contains additional genes of unknown function that are conserved in all toluene degraders, e.g., the *bssF* gene, or that are restricted to *bss* clusters from beta-proteobacteria, e.g., the *bssGIJKL* genes (**Figure 2**) (Kube et al., 2004; Carmona et al., 2009; Kim et al., 2014). To gain further knowledge on whether these additional *bss* genes might be essential for the initial reaction of the toluene/*m*-xylene degradation pathway, we constructed *Azoarcus* sp. CIB mutant strains with disruptional insertions within the *bssF* and *bssJ* genes. Whereas the strain *Azoarcus* sp. CIBd*bssF* was unable to grow anaerobically in toluene or *m*-xylene as sole carbon sources, the strain *Azoarcus* sp. CIBd*bssJ* could use these two aromatic hydrocarbons as growth substrates (**Table 1**). Therefore, these results reveal that inactivation of *bssF* prevents the anaerobic growth on toluene and *m*-xylene, and suggest that this gene and/or any of the downstream co-transcribed genes, i.e., *bssG, orfXY, bssI* (see below), are needed for the synthesis of a functional BSS enzyme. The BssF protein (580 amino acids) does not show significant similarity to other proteins of known function, which precludes so far any prediction of its functional role in the BSS activity. On the other hand, although the BssJ protein (307 amino acids) appears to be not essential for the BSS activity, we cannot rule out its involvement in toluene degradation by, for instance, mediating in the stress response caused by toxic aromatic hydrocarbons (Trautwein et al., 2008).

### Transcriptional Organization of the *bss-bbs* Gene Cluster

**FIGURE 4:**
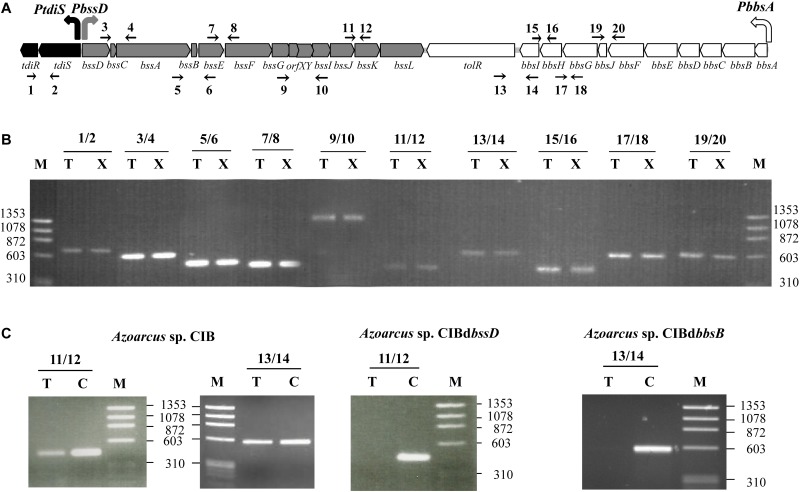
Transcriptional organization of the *tdi-bss-bbs* genes: **(A)** Schematic representation of the *tdi-bss-bbs* gene cluster from *Azoarcus* sp. CIB. The regulatory *tdi* genes are indicated by black arrows; the catabolic *bss* and *bbs* genes are indicated by gray and white arrows, respectively. The *PtdiS*, *PbssD* and *PbbsA* promoters are represented by bent arrows. The intergenic regions whose expression was checked by RT-PCR are marked by the oligonucleotides (thin arrows) used in the analysis (1–20; see Supplementary Table S1). **(B)** Agarose gel electrophoresis of RT-PCR products. RT-PCRs from *Azoarcus* sp. CIB cells grown under denitrifying conditions on 250 mM toluene (lanes T) or 250 mM *m*-xylene (lanes X) were performed as described in Section “Materials and Methods.” The numbers correspond to each primer pair that amplifies each of the intergenic regions indicated in **(A)**. Lanes M, molecular size markers (HaeIII-digested ΦX174 DNA); numbers indicate the sizes of the markers (in bp). **(C)** RT-PCRs from *Azoarcus* sp. CIB, *Azoarcus* sp. CIBd*bssD* and *Azoarcus* sp. CIBd*bbsB* cells anaerobically grown until stationary phase in 0.2% pyruvate plus 250 mM toluene (lanes T) by using the oligonucleotide pairs 11/12 and 13/14 (Supplementary Table S1). Lanes C, PCRs performed with the same primer pairs and with genomic DNA as a positive control. Lanes M, molecular size markers (HaeIII-digested ΦX174 DNA); numbers indicate the sizes of the markers (in bp).

The organization of the genes within the *bss-bbs* cluster in *Azoarcus* sp. CIB is very similar to that observed in other beta-proteobacteria (**Figure 2**). The *bss* and *bbs* genes are arranged in two opposite orientations and most of the genes are separated by very short distances suggesting that they constitute two convergent operons (Zamarro et al., 2016) (**Figure 4A**). The existence of the *bssDCABEFGH* catabolic operon has been described in “*A. aromaticum*” EbN1 (Kube et al., 2004), but it is not known whether these genes are cotranscribed with the *bssIJKLM* genes. On the contrary, whereas the *bss* cluster from *Azoarcus* sp. strain T contains a *bssDCABE* operon and a transcriptional initiation site upstream of *bssF* (Achong et al., 2001), the *tutE* (*bssD*) gene of *T. aromatica* T1 constitutes an operon different to that formed by *tutFDGH* (*bssCABE*) (Coschigano, 2000). On the other hand, the transcriptional organization of the *bbs* cluster has not been elucidated so far in any bacteria.

Since a transcriptional analysis of the whole gene cluster for anaerobic toluene and *m*-xylene degradation in bacteria has not been reported so far, we aimed to validate the presumed transcriptional organization of the *bss-bbs* genes in strain CIB. To this end, we performed RT-PCR experiments using total RNA harvested from *Azoarcus* sp. CIB cells grown anaerobically in toluene or *m*-xylene as sole carbon sources, and different primer sets that amplify *bssD-bssC* (3/4), *bssB-bssE* (5/6), *bssE-bssF* (7/8), *bssG-orfX-orfY-bssI* (9/10), *bssJ-bssK* (11/12), *tolR-bbsI* (13/14), *bbsI-bbsH* (15/16), *bbsH-bbsG* (17/18) and *bbsJ-bbsF* (19/20) gene fragments (**Figure 4A**). The results obtained (**Figure 4B**) strongly suggest that the *bssD-L* and *bbsA-tol* genes are co-transcribed and, therefore, constitute two separate convergent operons. To confirm that the *bss* and *bbs* genes constitute two separate operons, we performed gene expression studies in the *Azoarcus* sp. CIBd*bssD* and CIBd*bbsB* mutant strains that harbor insertional disruptions within the *bssD* and *bbsB* genes, respectively (**Table 1**). To this end, the two mutant strains were grown anaerobically in the presence of toluene, and RT-PCR analyses revealed the lack of expression of the *bss* and *bbs* genes (**Figure 4C**), hence suggesting that the mutations caused polar effects on the expression of the genes located dowsntream of the insertion site and, therefore, that these genes are co-transcribed. Therefore, all these data taken together suggest that in *Azoarcus* sp. CIB the *bssD-L* and *bbsA-tol* genes are arranged as two separate convergent operons. A comparative analysis of the upstream regions of the *bssD* and *bbsA* genes in different *Azoarcus, Aromatoleum, Thauera*, and *Herminiimonas* strains confirmed the existence of the previously proposed *PbssD* and *PbbsA* promoters, respectively (Supplementary Figure S1). *PbssD* and *PbbsA* contain potential -10/-35 sequences recognized by the RNA polymerase as well as the transcription initiation sites which have been mapped for *bssD* in *T. aromatica* (Coschigano, 2000; Hermuth et al., 2002; Kube et al., 2004) and *Azoarcus* sp. strain T (Achong et al., 2001), and for *bbsA* in *T. aromatica* K172 (Leuthner and Heider, 2000). Similar *PbssD* and *PbbsA* promoter regions driving the expression of the *bss* and *bbs* operons were identified in *Azoarcus* sp. CIB (**Figure 4A** and Supplementary Figure S1). Although an internal promoter or RNA processing event was reported upstream of *bssC* in some *Thauera* strains (Coschigano, 2000; Hermuth et al., 2002), the predicted stem-loop structure resembling a RNase processing site (Hermuth et al., 2002) is not observed in the *bss* operon of *Azoarcus* sp. CIB.

A couple of conserved genes, i.e., *tdiS* (*tutC1*) and *tdiR* (*tutB1*), are located upstream of the *bss* genes in most anaerobic toluene degraders from the beta-proteobacteria group (**Figure 2**). These two genes were proposed to code for a two-component regulatory system that regulates transcription of the catabolic *bss* genes (Coschigano, 2000; Achong et al., 2001; Hermuth et al., 2002; Kube et al., 2004; Kühner et al., 2005). A couple of orthologous *tdiSR* genes are also divergently transcribed with respect to the *bss* genes in *Azoarcus* sp. CIB (**Figures 1B**, **2**, **4A**). RT-PCR studies revealed that *tdiS* and *tdiR* are co-transcribed (**Figure 4B**) and, therefore, constitute an operon controlled by the *PtdiS* promoter in *Azoarcus* sp. CIB (**Figure 4A**). Moreover, the *tdiSR* operon appears to be slightly inducible when the cells grow anaerobically in toluene or *m*-xylene (**Figure 3C**), thus suggesting a common regulatory mechanism for the catabolic (*bss, bbs*) and predicted regulatory (*tdiSR*) genes in strain CIB.

All these results taken together constitute the first transcriptional analysis of the whole gene cluster responsible for the anaerobic peripheral pathway for toluene and *m*-xylene degradation in a single bacterial strain. In *Azoarcus* sp. CIB these genes are organized in two catabolic operons, *bssDCABEFGIJJKL* and *bbsABCDEFJGHtol*, and a putative regulatory operon, *tdiSR*, that are inducible when cells grow in toluene and *m*-xylene.

### *tdiSR* Control the Expression of the *bss-bbs* Genes in *Azoarcus* sp. CIB

To confirm the implication of the *tdiSR* genes in the anaerobic degradation of toluene and *m*-xylene in *Azoarcus* sp. CIB, we constructed two mutant strains, *Azoarcus* sp. CIBd*tdiS* and *Azoarcus* sp. CIBd*tdiR*, that harbor disruptional insertions within the *tdiS* (allows expression of *tdiR* from the kanamycin resistance gene, see Supplementary Figure S2) and *tdiR* genes, respectively. The two mutant strains were unable to grow using toluene or *m*-xylene as sole carbon sources (**Table 1**), thus suggesting that the *tdiSR* genes are involved in the anoxic degradation of toluene/*m*-xylene and they are likely behaving as an activator system of the *PbssD* and/or *PbbsA* promoters.

**FIGURE 5:**
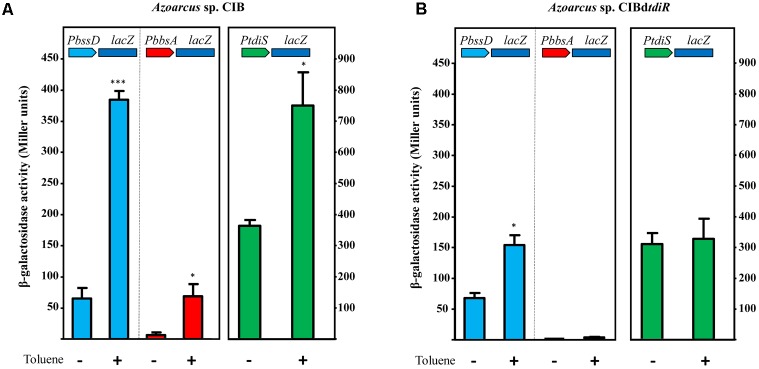
TdiR is a transcriptional activator of the *PbssD*, *PbbsA* and *PtdiS* promoters. Cells of *Azoarcus* sp. CIB **(A)** or *Azoarcus* sp. CIBd*tdiR* **(B)** containing plasmids pBBRPbssD, pBBRPbbsA, or pBBRPtdiS that express the *PbssD::lacZ*, *PbbsA::lacZ*, or *PtdiS::lacZ* translational fusions, respectively, were grown anaerobically on 0.2% pyruvate (–) or 0.2% pyruvate plus toluene (+) until stationary growth phase. β-galactosidase activity values were determined as detailed in Section “Materials and Methods.” Error bars represent standard deviation of three different experiments, and asterisks mark the results that are statistically significant (unpaired *t*-test; ^∗∗∗^*P*-value < 0.001, ^∗^*P*-value 0.01–0.05).

To study further the role of TdiSR in the activation of the *PbssD* and *PbbsA* promoters in *Azoarcus* sp. CIB, we constructed plasmids pBBRPbssD and pBBRPbbsA that contain the *PbssD::lacZ* and *PbbsA::lacZ* translational fusions, respectively (**Table 1**). The *Azoarcus* sp. CIB strains containing plasmid pBBRPbssD or pBBRPbbsA were grown anaerobically in the absence or presence of toluene or *m*-xylene, and β-galactosidase assays were performed at the end of the growth curve. As shown in **Figure 5A**, the presence of toluene increased the activity of the *PbssD* and *PbbsA* promoters by more than fivefold with respect to the activity observed in the absence of toluene (similar results were obtained with *m*-xylene, data not shown). These results are in agreement with the observed induction of the *bss* and *bbs* genes when *Azoarcus* sp. CIB grows anaerobically in toluene (**Figures 3A,B**). Moreover, the activity of the *PbssD* promoter appears to be significantly higher than that of the *PbbsA* promoter both in the absence or presence of toluene (**Figure 5A**). We then analyzed the activity of the *PbssD* and *PbbsA* promoters in the *Azoarcus* sp. CIBd*tdiR* mutant strain harboring the pBBRPbssD or pBBRPbbsA plasmids. In contrast to the wild-type strain, the *Azoarcus* sp. CIBd*tdiR* (pBBRPbbsA) strain did not show β-galactosidase activity even in the presence of toluene (**Figure 5B**), which strongly suggests that TdiR acts as an essential activator of the *PbbsA* promoter. On the other hand, the *Azoarcus* sp. CIBd*tdiR* (pBBRPbssD) strain revealed that the activity of the *PbssD* promoter region in the absence of toluene did not increase as much as in the wild-type strain in the presence of toluene (**Figure 5B**), suggesting that although TdiR is needed for a full induction of *bss* genes, there is some TdiR-independent activation of *PbssD* when the cells are in the presence of toluene.

Since the expression of the *tdiSR* genes is also induced when *Azoarcus* sp. CIB grows in the presence of toluene (**Figure 3C**), we checked whether the *tdiR* gene product could also act as a transcriptional activator of its own *PtdiS* promoter. To accomplish this, we constructed plasmid pBBRPtdiS, that contains the *PtdiS::lacZ* translational fusion (**Table 1**), and monitored the β-galactosidase activity in *Azoarcus* sp. CIB (pBBRPtdiS) and *Azoarcus* sp. CIBd*tdiR* (pBBRPtdiS) strains grown anaerobically in the absence or presence of toluene. Toluene increased the activity of the *PtdiS* promoter by more than twofold with respect to the activity observed in the absence of toluene in the wild-type but not in the *tdiR* mutant strain (**Figure 5**).

All these results taken together reveal for the first time that the *tdiSR* genes, which are highly conserved in the anaerobic toluene degradation clusters of beta-proteobacteria, encode an activator system of the promoters that drive expression of the *bss* and *bbs* catabolic genes and of that controlling transcription of the *tdi* regulatory genes in the presence of toluene. Whereas the expression of the *bbs* genes is strictly dependent on TdiR, the induction of the *bss* genes appears to be controlled by TdiR and some additional factors that respond to the presence of toluene in *Azoarcus* sp. CIB, and whose characterization requires further research.

### Benzylsuccinate Might Be the Inducer Molecule of the TdiSR-Mediated Control

**FIGURE 6:**
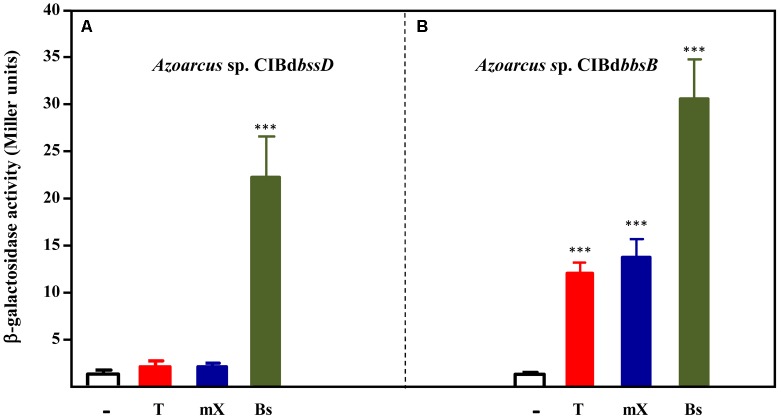
Analysis of the inducers of the *PbbsA* promoter. Cells of *Azoarcus* sp. CIBd*bssD* **(A)** or *Azoarcus* sp. CIBd*bbsB* **(B)** containing plasmid pBBRPbbsA that expresses the *PbbsA::lacZ* translational fusion, were grown anaerobically until stationary growth phase in 0.2% pyruvate (–) or in 0.2% pyruvate plus toluene (T), *m*-xylene (mX), or benzylsuccinate (Bs). β-galactosidase activity values were determined as detailed in Section “Materials and Methods.” Error bars represent standard deviation of three different experiments, and asterisks mark the results that are statistically significant (unpaired *t*-test; ^∗∗∗^*P*-value < 0.001).

As described above, the expression of the *tdi-bss-bbs* genes is inducible in *Azoarcus* sp. CIB cells growing in the presence of toluene and *m*-xylene (**Figures 3**, **4**). In *T. aromatica* T1, benzylsuccinate or any other further intermediate in the toluene peripheral pathway (**Figure 1A**), was suggested to be the inducer of the *tut*(*bss*) genes (Coschigano and Bishop, 2004). To determine if toluene and/or *m*-xylene, or an intermediate metabolite derived from the anaerobic degradation of the former, are the true inducers of the TdiSR-mediated control of the peripheral pathway in *Azoarcus* sp. CIB, we analyzed the expression of the *PbbsA::lacZ* fusion in different CIB strains. We selected CIB mutants strains that lack: (i) the first enzymatic step for the conversion of toluene/*m*-xylene to benzylsuccinate/(3-methyl)benzylsuccinate, i.e., *Azoarcus* sp. CIBd*bssD*, which does not express the *bss* operon (**Figure 4C**); (ii) the modified β-oxidation pathway that converts benzylsuccinate/(3-methyl)benzylsuccinate to the central intermediates benzoyl-CoA/3-methylbenzoyl-CoA (**Figure 1**), i.e., *Azoarcus* sp. CIBd*bbsB*, which does not express the *bbs* operon (**Figure 4C**). *Azoarcus* sp. CIBd*bssD* (pBBRPbbsA) cells grown anaerobically on pyruvate or pyruvate supplemented with toluene or *m*-xylene as inducers showed very low β-galactosidase activity in all the tested conditions, thus suggesting that none of the two aromatic hydrocarbons is the real inducer of the *bbs* operon (**Figure 6A**). On the contrary, *Azoarcus* sp. CIBd*bbsB* (pBBRPbbsA) cells, which retain a functional *bss* operon and should be capable to transform toluene and *m*-xylene accumulating benzylsuccinate and (3-methyl)benzylsuccinate, respectively, presented β-galactosidase activity when they were cultivated anaerobically in the presence of toluene or *m*-xylene (**Figure 6B**). Therefore, all these data suggest that benzylsuccinate and (3-methyl)benzylsuccinate, rather than toluene and *m*-xylene, are the real inducers of the *bbs* genes. In agreement with this hypothesis, the two *Azoarcus* sp. CIB mutant strains grown anaerobically in the presence of benzylsuccinate showed activation of the *PbbsA* promoter (**Figure 6**). Interestingly, none of *Azoarcus* strains could use benzylsuccinate as sole carbon and energy source, suggesting a poor uptake of this compound inside the cells.

Taken together all these results reveal that benzylsuccinate is the inducer compound of the TdiSR-mediated control of the toluene peripheral pathway in *Azoarcus* sp. CIB. Although a similar regulatory scenario can be predicted in other toluene degrader denitrifying bacteria, such as in *Thauera* strains (Coschigano and Bishop, 2004), benzylsuccinate may not always behave as the inducer molecule. Thus, it was reported that “*A. aromaticum*” EbN1 strain grown in a mixture of pyruvate and benzylsuccinate did not induce the expression of *bssA* or the formation of Bss/Bbs proteins (Kühner et al., 2005). Nevertheless, the lack of induction of benzylsuccinate in strain EbN1 could be due to unefficient transport of this molecule or to putative pyruvate-dependent catabolite repression of the *bss-bbs* genes, as it was shown in strain CIB (see below), and therefore further studies should be carried out to confirm whether benzylsuccinate behaves also as an inducer molecule in “*A. aromaticum*” EbN1.

**FIGURE 7:**
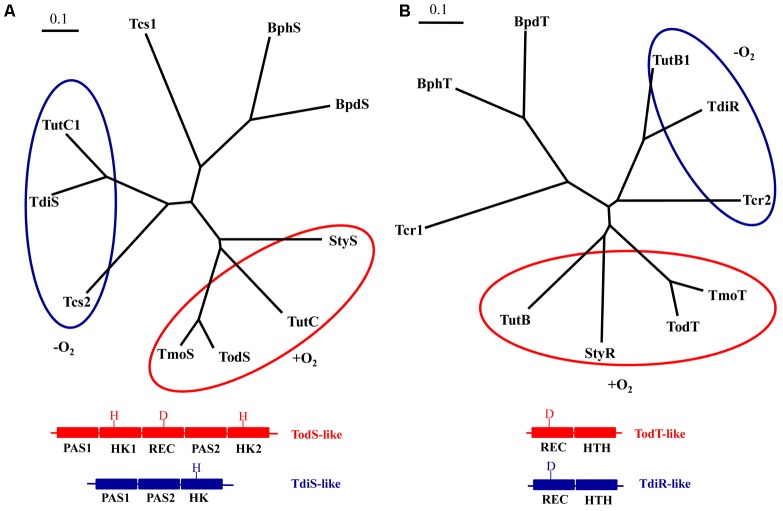
Phylogenetic relationships of the two-component regulatory systems involved in the regulation of aromatic hydrocarbon catabolic pathways: **(A)** Phylogenetic tree built from the multiple amino acid sequence alignment of the sensor histidine kinases TdiS from *Azoarcus* sp. CIB (ABK15651), TutC1 from *T. aromatica* T1 (AAD12187), Tcs2 from “*A. aromaticum*” sp. EbN1 (YP_158339), StyS from *Pseudomonas* sp. Y2 (CAA03998), TutC from *P. mendocina* (AAL13332), TodS from *P. putida* DOT-T1 (AAC45438), TmoS from *P. mendocina* (AAL13333), Tcs1 from *A. aromaticum* sp. EbN1 (YP_158337), BphS from *Rhodococcus* sp. RHA1 (BAC75411) and BpdS from *Rhodococcus* sp. M5 (AAB52543) using the program CLUSTALW and visualized with the TreeView software application. The aerobic TodS-like and anaerobic TdiS-like families are circled by red and blue lines, respectively, and a scheme of their different domain architecture and primary structure is shown at the bottom. PAS, HK, and REC correspond to the PAS sensor, autokinase and receiver domains, respectively. H and D indicate the presence of key phosphorylatable histidine and aspartic acid residues at the HK and REC domains, respectively. **(B)** Phylogenetic tree built from the multiple amino acid sequence alignment of the regulatory protein TdiR from *Azoarcus* sp. CIB (ABK15650), TutB1 from *T. aromatica* T1 (AAD12186), Tcr2 from “*A. aromaticum*” sp. EbN1 (YP_158340), TutB from *T. aromatica* T1 (AAD12186), StyR from *Pseudomonas* sp. Y2 (CAA03999), TodT from *P. putida* DOT-T1 (CAB43736), TmoT from *P. mendocina* (AAL13333), Tcr1 from *A. aromaticum* sp. EbN1 (YP_158338), BphT from *Rhodococcus* sp. RHA1 (BAC75412) and BpdT from *Rhodococcus* sp. M5 (AAB52544) using the program CLUSTALW and visualized with the TreeView software application. The aerobic TodT-like and anaerobic TdiR-like families are circled by red and blue lines, respectively, and a scheme of their similar domain architecture but different primary structure is shown at the bottom. REC and HTH correspond to the receiver and helix-turn-helix DNA-binding domains, respectively. D indicates the presence of key phosphorylatable aspartic acid residue at the REC domain. The bars represent one inferred amino acid substitution per ten amino acids.

Two-component regulatory systems that consist of a sensor histidine kinase and its cognate transcriptional regulator have been shown to be involved in the control of the catabolism of aromatic hydrocarbons in several bacteria (Carmona et al., 2008). According to their primary structure and molecular architecture, the sensor histidine kinases of these two-component regulatory systems can be classified in at least three different phylogenetic groups, i.e., the TodS, TdiS and BphS groups (**Figure 7A**). Hybrid histidine kinases of the TodS family are complex enzymes that contain two sensor PAS domains, i.e., PAS1, that recognizes aromatic hydrocarbons (Busch et al., 2007), and PAS2, involved in dimerization (Koh et al., 2016), two transmitter (autokinase) domains, and a response regulator receiver domain (**Figure 7A**) (Busch et al., 2009). They are involved in the control of the genes for the aerobic degradation of toluene in *Pseudomonas putida* (TodS) (Busch et al., 2007), *P. mendocina* (TmoS) (Silva-Jiménez et al., 2012) and, probably, in *T. aromatica* (TutC) (Leuthner and Heider, 1998), and styrene in some *Pseudomonas* strains (StyS) (Velasco et al., 1998; Leoni et al., 2003). Histidine kinases of the TdiS group are shorter than TodS-like kinases. They contain two tandem PAS domains, PAS1 and PAS2, and a single transmitter domain (**Figure 7A**), and are encoded in the clusters for the anaerobic degradation of toluene in some strains of the genera *Azoarcus*/“*Aromatoleum*” (TdiS) (Achong et al., 2001); *Thauera* (TdiS/TutC1) (Leuthner and Heider, 1998) and *Herminiimonas* (**Figure 2**), as well as in the cluster for the anaerobic degradation of ethylbenzene in “*A. aromaticum*” sp. EbN1 (Tcs2) (Kühner et al., 2005). On the other hand, the large histidine-kinases BphS and BpdS from *Rhodococcus* sp. RHA1 and *Rhodococcus* sp. M5, respectively, are involved in the aerobic degradation of biphenyls and they harbor a N-terminal domain similar to that of serine/threonine kinases and a C-terminal histidine-kinase domain (Takeda et al., 2010). It is worth noting that the amino acid residues shown to be involved in the binding to the inducer in the PAS1 domain of TodS, i.e., Phe46, Ile74, Phe79 and Ile114, are conserved in PAS1 domains of other TodS family members but not in any of the PAS domains of TdiS-like proteins. This observation is in agreement with the fact that TodS family members recognize aromatic hydrocarbons as inducers (Busch et al., 2007; Busch et al., 2009; Koh et al., 2016) but TdiS-like histidine kinases may respond to an intermediate, i.e., a benzylsuccinate derivative, rather than to the precursor aromatic hydrocarbon.

The response regulators of the two component regulatory systems that control the catabolism of aromatic hydrocarbons show a similar modular architecture with a N-terminal receiver domain that contains the phosphoaccepting Asp residue (putative Asp58 in TdiR) and a C-terminal helix-turn-helix (HTH) DNA-binding domain of the NarL/FixJ family separated by a glutamine-rich Q linker (**Figure 7B**) (Milani et al., 2005). As with the sensor histidine kinases, three different phylogenetic groups can be identified when comparing their cognate response regulator partners, i.e., the TodT, TdiR and BphT groups (**Figure 7B**). The 3D-structure of the HTH domain of StyR shows four α helices, one of them responsible of DNA recognition with three residues (Lys175, Val176 and His179) putatively involved in the interaction with the operator region of the cognate promoter (Milani et al., 2005). The same amino acid residues are conserved in other StyR-like regulators, such as TodT, TutB and TmoT, all of which may recognize the consensus ATAAACN_4_GTTTAT sequence at their corresponding operator regions (Lacal et al., 2006; Silva-Jiménez et al., 2012). Interestingly, these residues are not conserved in the HTH domains of the anaerobic TdiR-like response regulators, which were proposed to recognize a different palindromic region, GGTGTTCGCACC, that is conserved upstream of the -35 regions at the *PbssD* and *PbbsA* promoters of different anaerobic toluene degraders (Supplementary Figure S1) (Kube et al., 2004).

In *T. aromatica* T1 the genes *tutC1* (*tdiS*) and *tutB1* (*tdiR*) are separated from the *tutEFDGH (bssDCABE)* catabolic genes by *tutB* and *tutC* (**Figure 2**). The products of the *tutB* and *tutC* genes show high identity with TodS and TodT regulators, respectively, rather than with TdiS and TdiR regulators (**Figure 7**), thus strongly suggesting that TutBC are involved in the aerobic catabolism of toluene in *T. aromatica* T1 (Leuthner and Heider, 1998). However, the observation that a mutation in the *tutB* gene disabled the capacity to grow anaerobically in toluene (Coschigano and Young, 1997) and reduced significantly the expression of the *bss* genes (Coschigano and Bishop, 2004), might suggest the existence of some cross-talk in the regulation of the aerobic and anaerobic toluene degradation pathways in *T. aromatica* T1.

### Carbon Catabolite Repression of the Toluene/*m*-Xylene Peripheral Pathway in *Azoarcus* sp. CIB

The expression of the genes responsible of the catabolism of aromatic compounds is usually under carbon catabolite control when the cells grow in the presence of preferred carbon sources. This catabolite repression phenomenon has been extensively studied in the aerobic catabolism of aromatic compounds (Marqués et al., 1994; Cases et al., 1996; Carmona et al., 2008; Rojo, 2010), and it has also been described for the central pathways involved in the anaerobic catabolism of aromatic compounds in *T. aromatica* (Heider et al., 1998) and *Azoarcus* sp. CIB (López-Barragán et al., 2004; Carmona et al., 2009; Juárez et al., 2013; Valderrama et al., 2014). However, there were no reports about the expression of the peripheral pathways for anaerobic degradation of aromatic hydrocarbons when bacteria grow in the presence of alternative carbon sources.

**FIGURE 8:**
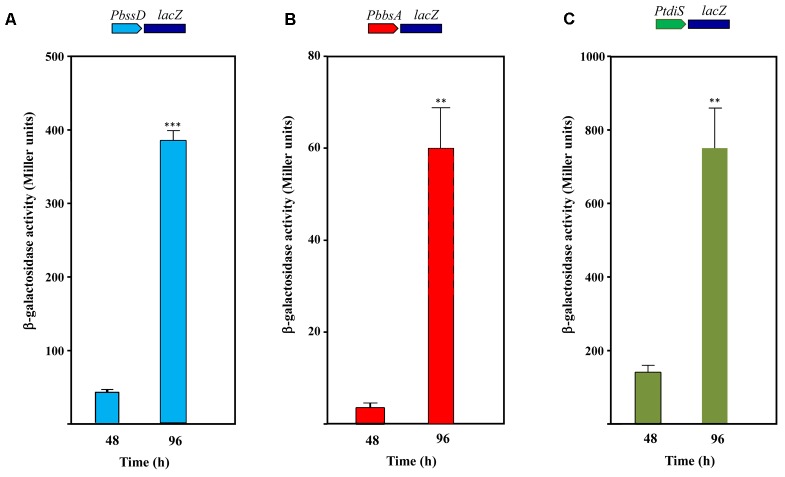
Carbon catabolite control of the *PbbsA*, *PbssD* and *PtdiS* promoters in *Azoarcus* sp. CIB. *Azoarcus* sp. CIB cells containing plasmid pBBRPbssD (*PbssD::lacZ* fusion) **(A)**, pBBRPbbsA (*PbbsA::lacZ* fusion) **(B)** or pBBRPtdiS (*PtdiS::lacZ* fusion) **(C)** were grown anaerobically in 0.2% pyruvate plus 250 mM toluene, and after 72 h they were supplemented with an additional amount of 10 mM nitrate. Culture samples were collected at mid-exponential phase (48 h) or at stationary phase (96 h). β-galactosidase activity values were determined as detailed in Section “Materials and Methods.” Error bars represent standard deviation of three different experiments, and asterisks mark the results that are statistically significant (unpaired *t*-test; ^∗∗∗^*P*-value < 0.001, ^∗∗^*P*-value 0.001–0.01).

To determine if the expression of the toluene/*m*-xylene anaerobic peripheral pathway in *Azoarcus* sp. CIB is under catabolite repression, we checked the activation of the *PbbsA* and *PbssD* promoters in *Azoarcus* sp. CIB (pBBRPbbsA) and *Azoarcus* sp. CIB (pBBRPbssD) cells, respectively, grown in minimal medium containing toluene plus an additional carbon source, e.g, pyruvate, glutamate, glutarate or benzoate. All the carbon sources tested produced an inhibitory effect on the induction of *PbbsA* and *PbssD* promoters, but this inhibition disappeared when the cells reached the stationary phase of growth and consumed the preferred carbon source (**Figures 8A,B**). In contrast, the induction of these promoters was observed at the exponential and stationary growth phases when the cells were grown in toluene as sole carbon source (Supplementary Figure S3). These results suggest that the *bss* and *bbs* genes are under carbon catabolite repression, but induction of these genes can be observed at the stationary growth phase as reported above (**Figures 5**, **6**). Interestingly, we also observed a pyruvate-dependent catabolite repression of the *PtdiS* promoter when *Azoarcus* sp. CIB (pBBRPtdiS) cells were grown in minimal medium containing toluene plus pyruvate, and this repression was alleviated at the stationary phase of growth (**Figure 8C**). Thus, catabolite repression of the *tdiST* regulatory operon might be a major reason of the observed repression by organic acids of the TdiSR-dependent expression of the *bss-bbs-*genes in *Azoarcus* sp. CIB. Although carbon catabolite repression by organic acids had been reported in the aerobic catabolism of toluene in some bacteria (Duetz et al., 1996; Ruíz et al., 2004; Busch et al., 2010), catabolite repression of the peripheral pathway for the anaerobic degradation of toluene has not been shown before.

### Evolutionary Considerations on the Toluene Peripheral Pathway

A detailed sequence comparison analysis of the organization of the *bss-bbs* genes in different beta-proteobacteria revealed the existence of a region that shows a high variability among strains. Thus, the *bssG-bssI* intergenic region contains a *bssH* gene encoding a putative transporter in “*A. aromaticum*” EbN1, but this gene is lacking in *Herminiimonas* sp. CN, and is substituted by *orfX-orfY*, two genes of unknown function, in *Azoarcus* sp. CIB and *A. toluclasticus* MF63 strains (**Figure 2**). Interestingly, this region divides the *bss-bbs* cluster of the CIB and MF63 strains into two different DNA fragments according to their GC content, i.e., a fragment containing the *bssDCABEFG* genes and whose GC content (58%) is lower that than of the fragment containing the rest of the *bss* (*bssIJKL*) and *bbs* genes, whose GC content (65%) matches the average GC content of the *Azoarcus* genome. It is worth noting that the *bssDCABE* genes from *Azoarcus* sp. T, *T. aromatica* T1 and *Thauera* sp. DNT-1 have also a GC content (<57%) lower than the average GC content (65%) described for *Azoarcus/Thauera* strains (Liu et al., 2013; Martín-Moldes et al., 2015). On the contrary, the GC content of the *bssDCABE* genes from “*A. aromaticum*” EbN1, *T. aromatica* K172 and *Magnetospirillum* sp. TS-6 is close to the average GC content of the corresponding species. These observations suggest that the *bssDCABEFG* genes from *Azoarcus* sp. CIB, *Azoarcus* sp. T, *T. aromatica* T1 and *Thauera* sp. DNT-1 have an evolutionary origin different than that of their orthologous from *Magnetospirillum* sp. TS-6, “*A. aromaticum*” EbN1 and T. *aromatica* K172, and most probably they come from a microorganism, such as a *Hermimiimonas*-related strain, with a GC content lower than that of *Thauera* or *Azoarcus* strains. Interestingly, this subgrouping of Bss orthologs has also a reflect in the subtle differences observed in their reaction mechanism when using stable isotope tools (Kummel et al., 2013).

Analysis of the flanking regions of the *tdi-bss-bbs* cluster in *Azoarcus* sp. CIB revealed the presence of full or truncated mobile genetic elements (**Figure 2**). Thus, next to the *tdiSR* genes there is a sequence (Δ*istA*) encoding the first amino acids of a truncated IS*21* transposase. *istA/istB* genes encoding the two subunits of a IS*21* transposase are located adjacent to the *bbs* genes in “*A. aromaticum*” EbN1 (**Figure 2**) (Kube et al., 2004). On the other hand, next to the *bbs* operon of strain CIB there is a set of genes (*orfB-E*) (**Figure 2**) that are orthologous to *c1A68*, *c1A87*, *c1A90*, and *c1A92* genes that encode proteins of unknown function and are located within the cluster for anaerobic degradation of ethylbenzene in “*A. aromaticum*” EbN1 strain (Rabus et al., 2002). These genes are also present in a similar organization next to the *bbs* genes in *A. toluclasticus* MF63 (**Figure 2**). Downstream of the *orfB-E* genes in strain CIB there are three additional genes encoding two complete and one truncated IS*4* transposase (**Figure 2**). All these observations strongly suggest that the *bss-bbs* genes in *Azoarcus* sp. CIB and some closely related bacteria have been acquired by horizontal gene transfer events as a result of different genetic rearrangements. A different genetic organization is observed in the *bss-bbs* clusters from phylogenetically distant delta-proteobacteria toluene-degrading obligate anaerobes, e.g., *Geobacter* or *Desulfobacula* (**Figure 2**) (Carmona et al., 2009; Wöhlbrand et al., 2013). The different genetic organization of the toluene peripheral pathway in facultative and obligate anaerobes correlates also with the significant differences found both in the predicted regulatory genes, e.g., TetR-like and XylR-like regulators are present in obligate anaerobes versus TdiSR-like regulators present in facultative anaerobes (**Figure 2**), and in the catabolic genes, e.g., *bss* and genes involved in auxiliary functions for Bss and Bbs enzymes are also distinct in facultative and strict anaerobes (**Figure 2**) (Wöhlbrand et al., 2013; Bozinovski et al., 2014). In summary, despite the biochemical strategy to convert toluene into benzoyl-CoA via benzylsuccinate appears to be shared by most anaerobic toluene-degraders, the complete set of genetic determinants involved in this peripheral pathway may have arisen through different evolutionary events in facultative and obligate anaerobes.

## Conclusion

Toluene and *m*-xylene are important contaminant hydrocarbons that tend to accumulate in subsurface anoxic environments. We have shown here that the *bss* and *bbs* genes are essential for the anaerobic degradation of toluene and *m*-xylene in *Azoarcus* sp. CIB. Moreover, we have characterized for the first time the transcriptional organization and regulation of a complete cluster encoding the peripheral pathway for the anaerobic degradation of toluene and *m*-xylene in bacteria. Benzylsuccinate and (3-methyl)benzylsuccinate were shown to be the inducer molecules recognized by the TdiSR two-component regulatory system that specifically controls the activation of the *bss*, *bbs* and *tdi* operons in *Azoarcus* sp. CIB. In addition to the TdiSR-mediated specific control, the expression of the *bss* and *bbs* genes in *Azoarcus* sp. CIB is under an overimposed regulation that depends on certain environmental factors, such as the presence/absence of oxygen or the availability of preferred carbon sources (catabolite repression). Interestingly, our results indicate that the *bss* and *bbs* operons from *Azoarcus* sp. CIB display differential regulation in the presence of toluene. Whereas the activity of the *PbbsA* promoter is strictly dependent on TdiR, the activation of the *PbssD* promoter is also under control of unknown factor(s) triggered by the presence of the aromatic hydrocarbons. Moreover, it is worth noting that the *PbssD* promoter shows a significant basal activity when the cells grow in the absence of toluene/*m*-xylene. This basal activity and the TdiR-independent activation of *PbssD* by toluene could be a strategy to increase the organism’s capacity to react quickly to transient toluene availability. Thus, the presence of basal levels of BSS enzyme in the cell will facilitate the formation of some inducer intermediate (benzylsuccinate) and, therefore, the subsequent full induction of all regulatory and catabolic genes when the cells face the presence of the aromatic hydrocarbons. Our results are in agreement with recent reports which suggest that *bssA* transcription may occur at a basal level even in the absence of toluene, supporting the idea that detection of *bssA* gene transcripts alone is not sufficient to indicate toluene degradation activity in contaminated environments (Brow et al., 2013; Lünsmann et al., 2016). In this context, and for a reliable assessment of microbial activity in toluene-contaminated samples, we propose here to monitor the expression of the tightly regulated *bbs* genes as an alternative or complementary approach to the current methods based only on the study of *bssA* expression. In this sense, highly conserved nucleotide sequence regions within the *bbsAB* genes of different bacteria could be used to design degenerate oligonucleotides primers for amplification of a *bbs* gene probe. Finally, the TdiSR-*PbbsA* regulatory couple identified in this work might constitute also an interesting genetic tool to develop whole cell biosensors for detecting benzylsuccinate, a widely used metabolic biomarker of *in situ* anaerobic bioremediation of toluene-contaminated sites (Young and Phelps, 2005).

## Author Contributions

BB carried out the practical work. BB, MC, and ED designed the experiments, analyzed the results, and wrote the manuscript.

## Conflict of Interest Statement

The authors declare that the research was conducted in the absence of any commercial or financial relationships that could be construed as a potential conflict of interest.

## Footnotes

1 http://www.ncbi.nlm.nih.gov

2 http://blast.ncbi.nlm.nih.gov

3 http://www.ebi.ac.uk/Tools/msa/clustalw2/

4 http://www.genebee.msu.su/services/phtree_reduced.html

## References

[B1] Achong G. R. Rodriguez A. M. Spormann A. M. (2001). Benzylsuccinate synthase of *Azoarcus* sp. strain T: cloning, sequencing, transcriptional organization, and its role in anaerobic toluene and *m*-xylene mineralization. *J. Bacteriol.* 183 6763–6770. 10.1128/JB.183.23.6763-6770.2001 11698363 PMC95515

[B2] Altschul S. Gish W. Miller W. Myers E. Lipman D. (1990). Basic local alignment search tool. *J. Mol. Biol.* 215 403–410. 10.1016/S0022-2836(05)80360-2 2231712

[B3] Beller H. R. Spormann A. M. (1998). Analysis of the novel benzylsuccinate synthase reaction for anaerobic toluene activation based on structural studies of the product. *J. Bacteriol.* 180 5454–5457. 9765580 10.1128/jb.180.20.5454-5457.1998 PMC107597

[B4] Bertani G. (1951). Studies on lysogenesis. I. The mode of phage liberation by lysogenic *Escherichia coli*. *J. Bacteriol.* 62 293–300. 14888646 10.1128/jb.62.3.293-300.1951 PMC386127

[B5] Bhandare R. Calabroa M. Coschigano P. W. (2006). Site-directed mutagenesis of the *Thauera aromatica* strain T1 *tutE tutFDGH* gene cluster. *Biochem. Biophys. Res. Commun.* 346 992–998. 10.1016/j.bbrc.2006.05.199 16780798

[B6] Blázquez B. Carmona M. García J. L. Díaz E. (2008). Identification and analysis of a glutaryl-CoA dehydrogenase-encoding gene and its cognate transcriptional regulator from *Azoarcus* sp. CIB. *Environ. Microbiol.* 10 474–482. 10.1111/j.1462-2920.2007.01468.x 18177371

[B7] Bozinovski D. Taubert M. Kleinsteuber S. Richnow H. H. von Bergen M. Vogt C. (2014). Metaproteogenomic analysis of a sulfate-reducing enrichment culture reveals genomic organization of key enzymes in the *m*-xylene degradation pathway and metabolic activity of proteobacteria. *Syst. Appl. Microbiol.* 37 488–501. 10.1016/j.syapm.2014.07.005 25156802

[B8] Brow C. N. O’Brien Johnson R. Johnson R. L. Simon H. M. (2013). Assessment of anaerobic toluene biodegradation activity by *bssA* transcript/gene ratios. *Appl. Environ. Microbiol.* 79 5338–5344. 10.1128/AEM.01031-13 23811506 PMC3753965

[B9] Busch A. Guazzaroni M. E. Lacal J. Ramos J. L. Krell T. (2009). The sensor kinase TodS operates by a multiple step phosphorelay mechanism involving two autokinase domains. *J. Biol. Chem.* 284 10353–10360. 10.1074/jbc.M900521200 19240030 PMC2667722

[B10] Busch A. Lacal J. Martos A. Ramos J. L. Krell T. (2007). Bacterial sensor kinase TodS interacts with agonistic and antagonistic signals. *Proc. Natl. Acad. Sci. U.S.A.* 104 13774–13779. 10.1073/pnas.0701547104 17693554 PMC1959458

[B11] Busch A. Lacal J. Silva-Jímenez H. Krell T. Ramos J. L. (2010). Catabolite repression of the TodS/TodT two-component system and effector-dependent transphosphorylation of TodT as the basis for toluene dioxygenase catabolic pathway control. *J. Bacteriol.* 192 4246–4250. 10.1128/JB.00379-10 20543072 PMC2916433

[B12] Butler J. E. He Q. Nevin K. P. He Z. Zhou J. Lovley D. R. (2007). Genomic and microarray analysis of aromatics degradation in *Geobacter metallireducens* and comparison to a *Geobacter* isolate from a contaminated field site. *BMC Genomics* 8:180. 10.1186/1471-2164-8-180 17578578 PMC1924859

[B13] Carmona M. Prieto M. A. Galán B. García J. L. Díaz E. (2008). “Signaling networks and design of pollutants biosensors,” in *Microbial Biodegradation: Genomics and Molecular Biology*, ed. Díaz E. (Norfolk, VA: Caister Academic Press), 97–143.

[B14] Carmona M. Zamarro M. T. Blázquez B. Durante-Rodríguez G. Juárez J. F. Valderrama J. A. (2009). Anaerobic catabolism of aromatic compounds: a genetic and genomic view. *Microbiol. Mol. Biol. Rev.* 73 71–133. 10.1128/MMBR.00021-08 19258534 PMC2650882

[B15] Cases I. de Lorenzo V. Pérez-Martín J. (1996). Involvement of sigma 54 in exponential silencing of the *Pseudomonas putida* TOL plasmid *Pu* promoter. *Mol. Microbiol.* 19 7–17. 10.1046/j.1365-2958.1996.345873.x 8821932

[B16] Chakraborty R. Coates J. D. (2004). Anaerobic degradation of monoaromatic hydrocarbons. *Appl. Microbiol. Biotechnol.* 64 437–446. 10.1007/s00253-003-1526-x 14735323

[B17] Chaurasia A. K. Tremblay P. L. Holmes D. E. Zhang T. (2015). Genetic evidence that the degradation of para-cresol by *Geobacter metallireducens* is catalyzed by the periplasmic *para*-cresol methylhydroxylase. *FEMS Microbiol. Lett.* 362:fnv145. 10.1093/femsle/fnv145 26316547

[B18] Coschigano P. W. (2000). Transcriptional analysis of the *tutE tutFDGH* gene cluster from *Thauera aromatica* strain T1. *Appl. Environ. Microbiol.* 66 1147–1151. 10.1128/AEM.66.3.1147-1151.2000 10698784 PMC91955

[B19] Coschigano P. W. Bishop B. J. (2004). Role of benzylsuccinate in the induction of the *tutE tutFDGH* gene complex of *T. aromatica* strain T1. *FEMS Microbiol. Lett.* 231 261–266. 10.1016/S0378-1097(04)00005-9 14987773

[B20] Coschigano P. W. Young L. Y. (1997). Identification and sequence analysis of two regulatory genes involved in anaerobic toluene metabolism by strain T1. *Appl. Environ. Microbiol.* 63 652–660. 9023943 10.1128/aem.63.2.652-660.1997 PMC168355

[B21] de Lorenzo V. Timmis K. N. (1994). Analysis and construction of stable phenotypes in gram-negative bacteria with Tn*5*- and Tn*10*-derived minitransposons. *Methods Enzymol.* 235 386–405. 10.1016/0076-6879(94)35157-0 8057911

[B22] Duetz W. A. Marqués S. Wind B. Ramos J. L. van Andel J. G. (1996). Catabolite repression of the toluene degradation pathway in *Pseudomonas putida* harboring pWW0 under various conditions of nutrient limitation in chemostat culture. *Appl. Environ. Microbiol.* 62 601–606. 8593060 10.1128/aem.62.2.601-606.1996 PMC167825

[B23] Durante-Rodríguez G. Zamarro M. T. García J. L. Díaz E. Carmona M. (2006). Oxygen-dependent regulation of the central pathway for the anaerobic catabolism of aromatic compounds in *Azoarcus* sp. strain CIB. *J. Bacteriol.* 188 2343–2354. 10.1128/JB.188.7.2343-2354.2006 16547020 PMC1428410

[B24] Felsenstein J. (1993). *PHYLIP (Phylogenetic Inference Package) Version 3.5.1.* Seattle, WA: University of Washington.

[B25] Fernández H. Prandoni N. Fernández-Pascual M. Fajardo S. Morcillo C. Díaz E. (2014). *Azoarcus* sp. CIB, an anaerobic biodegrader of aromatic compounds shows an endophytic lifestyle. *PLoS One* 9:e110771. 10.1371/journal.pone.0110771 25340341 PMC4207700

[B26] Fernández-Llamosas H. Castro L. Blázquez M. L. Díaz E. Carmona M. (2016). Biosynthesis of selenium nanoparticles by *Azoarcus* sp. CIB. *Microb. Cell Fact.* 15:109. 10.1186/s12934-016-0510-y 27301452 PMC4908764

[B27] Ferrández A. Miñambres B. García B. Olivera E. R. Luengo J. M. García J. L. (1998). Catabolism of phenylacetic acid in *Escherichia coli*. Characterization of a new aerobic hybrid pathway. *J. Biol. Chem.* 273 25974–25986. 10.1074/jbc.273.40.25974 9748275

[B28] Foght J. (2008). Anaerobic biodegradation of aromatic hydrocarbons: pathways and prospects. *J. Mol. Microbiol. Biotechnol.* 15 93–120. 10.1159/000121324 18685265

[B29] Funk M. A. Judd E. T. Marsh E. N. Elliott S. J. Drennan C. L. (2014). Structures of benzylsuccinate synthase elucidate roles of accessory subunits in glycyl radical enzyme activation and activity. *Proc. Natl. Acad. Sci. U.S.A.* 111 10161–10166. 10.1073/pnas.1405983111 24982148 PMC4104874

[B30] Heider J. Boll M. Breese K. Breinig S. Ebenau-Jehle C. Feil U. (1998). Differential induction of enzymes involved in anaerobic metabolism of aromatic compounds in the denitrifying bacterium *Thauera aromatica*. *Arch. Microbiol.* 170 120–131. 10.1007/s002030050623 9683649

[B31] Heider J. Szaleniec M. Martins B. M. Seyhan D. Buckel W. Golding B. T. (2016). Structure and function of benzylsuccinate synthase and related fumarate-adding glycyl radical enzymes. *J. Mol. Microbiol. Biotechnol.* 26 29–44. 10.1159/000441656 26959246

[B32] Hermuth K. Leuthner B. Heider J. (2002). Operon structure and expression of the genes for benzylsuccinate synthase in *Thauera aromatica* strain K172. *Arch. Microbiol.* 177 132–138. 10.1007/s00203-001-0375-1 11807562

[B33] Herrmann S. Vogt C. Fischer A. Kuppardt A. Richnow H. H. (2009). Characterization of anaerobic xylene biodegradation by two-dimensional isotope fractionation analysis. *Environ. Microbiol. Rep.* 1 535–544. 10.1111/j.1758-2229.2009.00076.x 23765932

[B34] Juárez J. F. Liu H. Zamarro M. T. McMahon S. Liu H. Naismith J. H. (2015). Unraveling the specific regulation of the central pathway for anaerobic degradation of 3-methylbenzoate. *J. Biol. Chem.* 290 12165–12183. 10.1074/jbc.M115.637074 25795774 PMC4424350

[B35] Juárez J. F. Zamarro M. T. Eberlein C. Boll M. Carmona M. Díaz E. (2013). Characterization of the *mbd* cluster encoding the anaerobic 3-methylbenzoyl-CoA central pathway. *Environ. Microbiol.* 15 148–166. 10.1111/j.1462-2920.2012.02818.x 22759228

[B36] Kim S. J. Park S. J. Jung M. Y. Kim J. G. Madsen E. L. Rhee S. K. (2014). An uncultivated nitrate-reducing member of the genus *Herminiimonas* degrades toluene. *Appl. Environ. Microbiol.* 80 3233–3243. 10.1128/AEM.03975-13 24632261 PMC4018906

[B37] Kimura M. (1980). A simple method for estimating evolutionary rates of base substitutions through comparative studies of nucleotide sequences. *J. Mol. Evol.* 16 111–120. 10.1007/BF01731581 7463489

[B38] Koh S. Hwang J. Guchhait K. Lee E. G. Kim S. Y. Kim S. (2016). Molecular insights into toluene sensing in the TodS/TodT signal transduction system. *J. Biol. Chem.* 291 8575–8590. 10.1074/jbc.M116.718841 26903514 PMC4861429

[B39] Krieger C. J. Beller H. R. Reinhard M. Spormann A. M. (1999). Initial reactions in anaerobic oxidation of *m*-xylene by the denitrifying bacterium *Azoarcus* sp. strain T. *J. Bacteriol.* 181 6403–6410. 10515931 10.1128/jb.181.20.6403-6410.1999 PMC103776

[B40] Krieger C. J. Roseboom W. Albracht S. P. Spormann A. M. (2001). A stable organic free radical in anaerobic benzylsuccinate synthase of *Azoarcus* sp. strain T. *J. Biol. Chem.* 276 12924–12927. 10.1074/jbc.M009453200 11278506

[B41] Kube M. Heider J. Amann J. Hufnagel P. Kühner S. Beck A. (2004). Genes involved in the anaerobic degradation of toluene in a denitrifying bacterium, strain EbN1. *Arch. Microbiol.* 181 182–194. 10.1007/s00203-003-0627-3 14735297

[B42] Kühner S. Wöhlbrand L. Fritz I. Wruck W. Hultschig C. Hufnagel P. (2005). Substrate-dependent regulation of anaerobic degradation pathways for toluene and ethylbenzene in a denitrifying bacterium, strain EbN1. *J. Bacteriol.* 187 1493–1503. 10.1128/JB.187.4.1493-1503.2005 15687214 PMC545613

[B43] Kummel S. Kuntze K. Vogt C. Boll M. Heider J. Richnow H. H. (2013). Evidence for benzylsuccinate synthase subtypes obtained by using stable isotope tools. *J. Bacteriol.* 195 4660–4667. 10.1128/JB.00477-13 23935041 PMC3807448

[B44] Lacal J. Busch A. Guazzaroni M. E. Krell T. Ramos J. L. (2006). The TodS-TodT two-component regulatory system recognizes a wide range of effectors and works with DNA-bending proteins. *Proc. Natl. Acad. Sci. U.S.A.* 103 8191–8196. 10.1073/pnas.0602902103 16702539 PMC1472451

[B45] Leoni L. Ascenzi P. Bocedi A. Rampioni G. Castellini L. Zennaro E. (2003). Styrene-catabolism regulation in *Pseudomonas fluorescens* ST: phosphorylation of StyR induces dimerization and cooperative DNA-binding. *Biochem. Biophys. Res. Commun.* 303 926–931. 10.1016/S0006-291X(03)00450-9 12670500

[B46] Leuthner B. Heider J. (1998). A two-component system involved in regulation of anaerobic toluene metabolism in *Thauera aromatica*. *FEMS Microbiol. Lett.* 166 35–41. 10.1111/j.1574-6968.1998.tb13180.x 9741082

[B47] Leuthner B. Heider J. (2000). Anaerobic toluene catabolism of *Thauera aromatica:* the *bbs* operon codes for enzymes of β oxidation of the intermediate benzylsuccinate. *J. Bacteriol.* 182 272–277. 10.1128/JB.182.2.272-277.2000 10629170 PMC94273

[B48] Leuthner B. Leutwein C. Schulz H. Hörth P. Haehnel W. Schiltz E. (1998). Biochemical and genetic characterization of benzylsuccinate synthase from *Thauera aromatica*: a new glycyl radical enzyme catalysing the first step in anaerobic toluene metabolism. *Mol. Microbiol.* 28 615–628. 10.1046/j.1365-2958.1998.00826.x 9632263

[B49] Leutwein C. Heider J. (2001). Succinyl-CoA:(R)-benzylsuccinate CoA-transferase: an enzyme of the anaerobic toluene catabolic pathway in denitrifying bacteria. *J. Bacteriol.* 183 4288–4295. 10.1128/JB.183.14.4288-4295.2001 11418570 PMC95319

[B50] Leutwein C. Heider J. (2002). (R)-Benzylsuccinyl-CoA dehydrogenase of *Thauera aromatica*, an enzyme of the anaerobic toluene catabolic pathway. *Arch. Microbiol.* 178 517–524. 10.1007/s00203-002-0484-5 12420174

[B51] Liu B. Frostegård A. Shapleigh J. P. (2013). Draft genome sequences of five strains in the genus *Thauera*. *Genome Announc.* 1:e00052–12 10.1128/genomeA.00052-12 PMC3569365 23405361

[B52] López-Barragán M. J. Carmona M. Zamarro M. T. Thiele B. Boll M. Fuchs G. (2004). The bzd gene cluster, coding for anaerobic benzoate catabolism, in *Azoarcus* sp. strain CIB. *J. Bacteriol.* 186 5762–5774. 10.1128/JB.186.17.5762-5774.2004 15317781 PMC516837

[B53] Lovley D. R. Baedecker M. J. Lonergan D. J. Cozzarelli I. M. Phillips E. J. P. Siegel D. I. (1989). Oxidation of aromatic contaminants coupled to microbial iron reduction. *Nature* 339 297–300. 10.1038/339297a0 18075087

[B54] Lueders T. (2017). The ecology of anaerobic degraders of BTEX hydrocarbons in aquifers. *FEMS Microbiol. Ecol.* 93:fiw220. 10.1093/femsec/fiw220 27810873 PMC5400083

[B55] Lünsmann V. Kappelmeyer U. Taubert A. Nijenhuis I. von Bergen M. Heipieper H. J. (2016). Aerobic toluene degraders in the rhizosphere of a constructed wetland model show diurnal polyhydroxyalkanoate metabolism. *Appl. Environ. Microbiol.* 82 4126–4132. 10.1128/AEM.00493-16 27129963 PMC4959214

[B56] Marqués S. Holtel A. Timmis K. N. Ramos J. L. (1994). Transcriptional induction kinetics from the promoters of the catabolic pathways of TOL plasmid pWW0 of *Pseudomonas putida* for metabolism of aromatics. *J. Bacteriol.* 176 2517–2524. 10.1128/jb.176.9.2517-2524.1994 8169200 PMC205388

[B57] Martín-Moldes Z. Blázquez B. Baraquet C. Harwood C. S. Zamarro M. T. Díaz E. (2016). Degradation of cyclic diguanosine monophosphate by a hybrid two-component protein protects *Azoarcus* sp. strain CIB from toluene toxicity. *Proc. Natl. Acad. Sci. U.S.A.* 113 13174–13179. 10.1073/pnas.1615981113 27799551 PMC5135311

[B58] Martín-Moldes Z. Zamarro M. T. del Cerro C. Valencia A. Gómez M. J. Arcas A. (2015). Whole-genome analysis of *Azoarcus* sp. strain CIB provides genetic insights to its different lifestyles and predicts novel metabolic features. *Syst. Appl. Microbiol.* 38 462–471. 10.1016/j.syapm.2015.07.002 26259823

[B59] Meyer-Cifuentes I. Martinez-Lavanchy P. M. Marin-Cevada V. Böhnke S. Harms H. Müller J. A. (2017). Isolation and characterization of *Magnetospirillum* sp. strain 15-1 as a representative anaerobic toluene-degrader from a constructed wetland model. *PLoS One* 12:e0174750. 10.1371/journal.pone.0174750 28369150 PMC5378359

[B60] Milani M. Leoni L. Rampioni G. Zennaro E. Ascenzi P. Bolognesi M. (2005). An active-like structure in the unphosphorylated StyR response regulator suggests a phosphorylation- dependent allosteric activation mechanism. *Structure* 13 1289–1297. 10.1016/j.str.2005.05.014 16154086

[B61] Miller J. H. (1972). *Experiments in Molecular Genetics.* New York, NY: Cold Spring Harbord Laboratory.

[B62] Morasch B. Schink B. Tebbe C. C. Meckenstock R. U. (2004). Degradation of o-xylene and m-xylene by a novel sulfate-reducer belonging to the genus *Desulfotomaculum*. *Arch. Microbiol.* 181 407–417. 10.1007/s00203-004-0672-6 15127183

[B63] Rabus R. Boll M. Heider J. Meckenstock R. U. Buckel W. Einsle O. (2016). Anaerobic microbial degradation of hydrocarbons: from enzymatic reactions to the environment. *J. Mol. Microbiol. Biotechnol.* 26 5–28. 10.1159/000443997 26960061

[B64] Rabus R. Kube M. Beck A. Widdel F. Reinhardt R. (2002). Genes involved in the anaerobic degradation of ethylbenzene in a denitrifying bacterium, strain EbN1. *Arch. Microbiol.* 178 506–516. 10.1007/s00203-002-0487-2 12420173

[B65] Rojo F. (2010). Carbon catabolite repression in *Pseudomonas*: optimizing metabolic versatility and interactions with the environment. *FEMS Microbiol. Rev.* 34 658–684. 10.1111/j.1574-6976.2010.00218.x 20412307

[B66] Ruíz R. Aranda-Olmedo M. I. Domínguez-Cuevas P. Ramos-González M. I. Marqués S. (2004). “Transcriptional regulation of the toluene catabolic pathways,” in *Pseudomonas Virulence and Gene Regulation* Vol. 2 ed. Ramos J. L. (New York, NY: Kluwer Academic), 509–537.

[B67] Saitou N. Nei M. (1987). The neighbor-joining method: a new method for reconstructing phylogenetic trees. *Mol. Biol. Evol.* 4 406–425. 3447015 10.1093/oxfordjournals.molbev.a040454

[B68] Sambrook J. Russell D. W. (2001). *Molecular Cloning: A Laboratory Manual.* New York, NY: Cold Spring Harbor.

[B69] Sanger F. Nicklen S. Coulson A. R. (1977). DNA sequencing with chain-terminating inhibitors. *Proc. Natl. Acad. Sci. U.S.A.* 74 5463–5467. 10.1073/pnas.74.12.5463 271968 PMC431765

[B70] Schäfer A. Tauch A. Jäger W. Kalinowski J. Thierbach G. Pühler A. (1994). Small mobilizable multi-purpose cloning vectors derived from the *Escherichia coli* plasmids pK18 and pK19: selection of defined deletions in the chromosome of *Corynebacterium glutamicum*. *Gene* 145 69–73. 10.1016/0378-1119(94)90324-7 8045426

[B71] Selmer T. Pierik A. J. Heider J. (2005). New glycyl radical enzymes catalysing key metabolic steps in anaerobic bacteria. *Biol. Chem.* 386 981–988. 10.1515/BC.2005.114 16218870

[B72] Shinoda Y. Akagi J. Uchihashi Y. Hiraishi A. Yukawa H. Yurimoto H. (2005). Anaerobic degradation of aromatic compounds by *Magnetospirillum* strains: isolation and degradation genes. *Biosci. Biotechnol. Biochem.* 69 1483–1491. 10.1271/bbb.69.1483 16116275

[B73] Shinoda Y. Sakai Y. Uenishi H. Uchihashi Y. Hiraishi A. Yukawa H. (2004). Aerobic and anaerobic toluene degradation by a newly isolated denitrifying bacterium, *Thauera* sp. strain DNT-1. *Appl. Environ. Microbiol.* 70 1385–1392. 10.1128/AEM.70.3.1385-1392.2004 15006757 PMC368410

[B74] Silva-Jiménez H. García-Fontana C. Cadirci B. H. Ramos-González M. I. Ramos J. L. Krell T. (2012). Study of the TmoS/TmoT two-component system: towards the functional characterization of the family of TodS/TodT like systems. *Microb. Biotechnol.* 5 489–500. 10.1111/j.1751-7915.2011.00322.x 22212183 PMC3815326

[B75] Strijkstra A. Trautwein K. Jarling R. Wöhlbrand L. Dörries M. Reinhardt R. (2014). Anaerobic activation of *p*-cymene in denitrifying betaproteobacteria: methyl group hydroxylation versus addition to fumarate. *Appl. Environ. Microbiol.* 80 7592–7603. 10.1128/AEM.02385-14 25261521 PMC4249252

[B76] Takeda H. Shimodaira J. Yukawa K. Hara N. Kasai D. Miyauchi K. (2010). Dual two-component regulatory systems are involved in aromatic compound degradation in a polychlorinated-biphenyl degrader, *Rhodococcus jostii* RHA1. *J. Bacteriol.* 192 4741–4751. 10.1128/JB.00429-10 20622058 PMC2937422

[B77] Thompson J. D. Higgins D. G. Gibson T. J. (1994). CLUSTAL W: improving the sensitivity of progressive multiple sequence alignment through sequence weighting, position-specific gap penalties and weight matrix choice. *Nucleic Acids Res.* 22 4673–4680. 10.1093/nar/22.22.4673 7984417 PMC308517

[B78] Trautwein K. Kühner S. Wöhlbrand L. Halder T. Kuchta K. Steinbüchel A. (2008). Solvent stress response of the denitrifying bacterium “*Aromatoleum aromaticum*” strain EbN1. *Appl. Environ. Microbiol.* 74 2267–2274. 10.1128/AEM.02381-07 18263750 PMC2293168

[B79] Valderrama J. A. Durante-Rodríguez G. Blázquez B. García J. L. Carmona M. Díaz E. (2012). Bacterial degradation of benzoate: cross-regulation between aerobic and anaerobic pathways. *J. Biol. Chem.* 287 10494–10508. 10.1074/jbc.M111.309005 22303008 PMC3322966

[B80] Valderrama J. A. Shingler V. Carmona M. Díaz E. (2014). AccR is a master regulator involved in carbon catabolite repression of the anaerobic catabolism of aromatic compounds in *Azoarcus* sp. CIB. *J. Biol. Chem.* 289 1892–1894. 10.1074/jbc.M113.517714 24302740 PMC3900940

[B81] Velasco A. Alonso S. García J. L. Perera J. Díaz E. (1998). Genetic and functional analysis of the styrene catabolic cluster of *Pseudomonas* sp. Strain Y2. *J. Bacteriol.* 180 1063–1071. 9495743 10.1128/jb.180.5.1063-1071.1998 PMC106992

[B82] Verfürth K. Pierik A. J. Leutwein C. Zorn S. Heider J. (2004). Substrate specificities and electron paramagnetic resonance properties of benzylsuccinate synthases in anaerobic toluene and m-xylene metabolism. *Arch. Microbiol.* 181 155–162. 10.1007/s00203-003-0642-4 14689166

[B83] Wöhlbrand L. Jacob J. H. Kube M. Mussmann M. Jarling R. Beck A. (2013). Complete genome, catabolic sub-proteomes and key-metabolites of *Desulfobacula toluolica* Tol2 a marine, aromatic compound-degrading, sulfate-reducing bacterium. *Environ. Microbiol.* 15 1334–1355. 10.1111/j.1462-2920.2012.02885.x 23088741

[B84] Young L. Y. Phelps C. D. (2005). Metabolic biomarkers for monitoring in situ anaerobic hydrocarbon degradation. *Environ. Health Perspect.* 113 62–67. 10.1289/ehp.6940 15626649 PMC1253711

[B85] Zamarro M. T. Martín-Moldes Z. Díaz E. (2016). The ICEXTD of *Azoarcus* sp. CIB, an integrative and conjugative element with aerobic and anaerobic catabolic properties. *Environ. Microbiol.* 18 5018–5031. 10.1111/1462-2920.13465 27450529

