# Supplementary material for: Transcriptional Regulation of the Peripheral Pathway for the Anaerobic Catabolism of Toluene and m-Xylene in Azoarcus sp. CIB
Source: Front Microbiol. 2018 Mar 22;9:506. doi: 10.3389/fmicb.2018.00506 (PMC5874301; doi:10.3389/fmicb.2018.00506)
Supplement: Supplementary file 1 [file Image_1.PDF]

SUPPLEMENTAL MATERIAL

Transcriptional Regulation of the Peripheral Pathway for the Anaerobic Catabolism of Toluene and *m*-Xylene in *Azoarcus* sp. CIB

Blas Blázquez[1], Manuel Carmona and Eduardo Díaz*

Department of Microbial and Plant Biotechnology, Centro de Investigaciones Biológicas-CSIC, Ramiro de Maeztu 9, 28040 Madrid, Spain

*** Correspondence**

Dr. Eduardo Díaz

ediaz@cib.csic.es

[1]Present address

Systems Biology Programme, Centro Nacional de Biotecnología-CSIC, Darwin 3, 28049 Madrid, Spain

TABLE S1 Oligonucleotides used in this study

Primers	Sequence (restriction site)	Use
5PbbsA	CCTCTAGACGACCGGACAGCTCAAGGCGC (XbaI)	356-bp *orfB-bbsA* intergenic fragment including *PbbsA* promoter
3PbbsA	AACTGCAGGACATGACGCCTCCGCAGCATTTG (PstI)	356-bp *orfB-bbsA* intergenic fragment including *PbbsA* promoter
5PtdiS	GGACTAGTCACCGCGACCACGTCATTCTTCATG (SpeI)	335-bp *ptdiS-bssD* intergenic fragment including *PbssD* promoter
3PbssD	CGGGATCCCTCATGACAGCCTCTGCGGTTCATTGC (BamHI)	335-bp *ptdiS-bssD* intergenic fragment including *PbssD* promoter & 314-bp *bssD-tdiS* intergenic fragment including *PtdiS* promoter
PtdiS3´	CGGGATCCTTCATGGAGCACTACCTCCTCGCAACC	314-bp *bssD-tdiS* intergenic fragment including *PtdiS* promoter
1(tdiRint5)	CACGGTTTTGGGGCTGATCC	400-bp *tdiR* internal fragment cloned into pK18*mob* to generate pK18*mobtdiR* & amplification of 680-bp fragment for RT-PCR
tdiR131.5	GCTCTAGACGACGCGGATATTTCAGGTTCCTACGG (XbaI)	400-bp *tdiR* internal fragment cloned into pK18*mob* to generate pK18*mobtdiR*
5TdiS	CATCTCCGAAACCGCACGATCAAGC	623-bp *tdiS* internal fragment cloned into pK18*mob* to generate pK18*mobtdiS*
3TdiS	GATCTCCGTTCCGTCGTGCAGG	623-bp *tdiS* internal fragment cloned into pK18*mob* to generate pK18*mobtdiS*
5BssD	AACTGCAGGCCTGACGGACGCGCGCACC (PstI)	593-bp *bssD* internal fragment cloned into pK18*mob* to generate pK18*mobbssD*
3BssD	CGGGATCCGCCAATCGTTACGTTCTTGATGCCTGC (BamHI)	593-bp *bssD* internal fragment cloned into pK18*mob* to generate pK18*mobbssD*
5BssF	CACAAGCTTGAGCAAGTGGTGCGG (HindIII)	451-bp *bssF* internal fragment cloned into pK18*mob* to generate pK18*mobbssF*
3BssF	GGTCTAGAGAGGCGCCGTCGGTGATGTG (XbaI)	451-bp *bssF* internal fragment cloned into pK18*mob* to generate pK18*mobbssF*
5BssJ	AACTGCAGCGGGCCTACGAGCG (PstI)	347-bp *bssJ* internal fragment cloned into pK18*mob* to generate pK18*mobbssJ*
3BssJ	AAGTCGACCCACGTGCGAGCACGCGGGC (SalI)	347-bp *bssJ* internal fragment cloned into pK18*mob* to generate pK18*mobbssJ*
5BbsB	CCGAGGATCTGGCGATGATCACCG	512-bp *bbsB* internal fragment cloned into pK18*mob* to generate pK18*mobbbsB*
3BbsB	AAGGATCCCCCGTAACGTGCGTCATCGCCAC (BamHI)	512-bp *bbsB* internal fragment cloned into pK18*mob* to generate pK18*mobbbsB*
bssA5new	CGCTCAATTTCACACCTGAAGATC	Amplification of 575-bp fragment *bssA* for RT-PCR
bssA3new	CAAGGACGGTTTCGTAGGGCGGTAC	Amplification of 575-bp fragment *bssA* for RT-PCR
bbsA331.3	GGTTGTCGCGAACGGGCCCC	Amplification of 290-bp fragment *bbsA* for RT-PCR
bbsA5new	ATCTCAAGGGGTATCGCTGCAAGG	Amplification of 290-bp fragment *bbsA* for RT-PCR
2 (tdiSF.3)	GTCGAGTCGCACGGCGGGCAGC	Amplification of 680-bp fragment for RT-PCR
3 (3T7.3)	CATGCAGCTGGAGGGTGTCG	Amplification of 582-bp fragment for RT-PCR
4 (bsssAint3)	GATGCTCGTGCAACTCGTCGGATGG	Amplification of 582-bp fragment for RT-PCR
5 (3T7.7)	GACTTCAGCGCGTCCGATCTCG	Amplification of 449-bp fragment for RT-PCR
6 (bssEint3)	GGGGTCCACTTCCGGGATGAACAAGCC	Amplification of 449-bp fragment for RT-PCR
7 (3T7.9)	CGAATACGTTCAGTACATTGCCGC	Amplification of 408-bp fragment for RT-PCR
8 (bssFint3)	GGACGCGCAGCCTGAGCATCTCG	Amplification of 408-bp fragment for RT-PCR

9 (bssGint5)	CGCCGCCAATCGCAATCTCGC	Amplification of 1220-bp fragment for RT-PCR
10 (3T3.14)	CGTGACGACAAGCCCGCCATC	Amplification of 1220-bp fragment for RT-PCR
11 (c2A200int5)	CTCGTCTACCTGCTGAACCGCCGC	Amplification of 404-bp fragment for RT-PCR
12 (3T3.12)	CGGCGCCGGGCATCTTCAGC	Amplification of 404-bp fragment for RT-PCR
13 (TolSRint3)	GCCGTCGAGGATGATGCGTGCC	Amplification of 564-bp fragment for RT-PCR
14 (3T3.3)	CGAGCACATGGCGGGGCTGTAC	Amplification of 564-bp fragment for RT-PCR
15 (bbsIint3)	CGGCGTTCGGCGTCCACCACC	Amplification of 397-bp fragment for RT-PCR
16 (3T3.2)	CGGGCTTGTCAGCGACGTGG	Amplification of 397-bp fragment for RT-PCR
17 (3bbsH333)	CATCTCGAGCCCGCCACCCACC	Amplification of 563-bp fragment for RT-PCR
18 (bbsG.F5)	GCTCACGCGTGTCGCCGACCG	Amplification of 563-bp fragment for RT-PCR
19 (bbsGint3)	GTGAACCGCCGGGCCACCTCC	Amplification of 592-bp fragment for RT-PCR
20 (bbsFint5)	GGAAATGAAGACCGCTGCGCCG	Amplification of 592-bp fragment for RT-PCR
5polIIIHK	CGAAACGTCGGCATGCACG	220-bp internal fragment of housekeeping gene *dnaE* (DNApol III α subunit)
3polIIIHK	GCGCAGGCCTAGGAAGTCGAAC	220-bp internal fragment of housekeeping gene *dnaE* (DNApol III α subunit)

Supplemental Figure Legends

FIGURE S1. Alignment of the *PbssD* and *PbbsA* promoter sequences from different bacteria. The known or predicted transcription initiation sites (+1) and -10/-35 sequences of interaction with the σ^{70}-RNA polymerase are indicated in grey. The predicted operator sequences recognized by the TdiR transcriptional regulator are boxed. Numbers indicated the distance (in nucleotides) to the GTG and ATG start codon of *bssD* or *bbsA* genes, respectively. The accession numbers of the nucleotide sequences are those indicated in Figure 2. Modified from: Kube, M., Heider, J., Amann, J., Hufnagel, P, Kühner, S., Beck. A., Reinhardt. R., and Rabus, R. (2004) Genes involved in the anaerobic degradation of toluene in a denitrifying bacterium, strain EbN1. *Arch Microbiol* 181, 182-194.

FIGURE S2. The *tdiR* gene is transcribed in *Azoarcus* sp. CIBd*tdiS* cells. Agarose gel electrophoresis of RT-PCR products. RT-PCRs from *Azoarcus* sp. CIBd*tdiS* cells grown under denitrifying conditions on pyruvate + toluene (lane T) or pyruvate + *m*-xylene (lane X) were performed as described in Materials and Methods with the primer pair 1/2 (Supplementary Table S1) that amplifies a *tdiS*-*tdiR* intergenic fragment. Lanes C, PCRs performed with the same primer pair and with RNA as negative control. Lane M, molecular size markers (HaeIII-digested FX174 DNA); numbers indicate the sizes of the markers (in bp).

FIGURE S3. Expression of the *PbbsA::lacZ* translational fusion in *Azoarcus* sp. CIB cells grown anaerobically in toluene. *Azoarcus* sp. CIB cells containing plasmid pBBRPbbsA that expresses the *PbbsA::lacZ* translational fusion, were grown anaerobically in toluene and samples were taken at the exponential (48h) and stationary (96h) growth phases. β-galactosidase activity values were determined as detailed in Materials and Methods. Error bars represent standard deviation of three different experiments.

***PbssD* promoter**

Azoarcus sp. CIB	CCGC-ACTA-GGGACAACCCCTCTTTGCTTAAGTGTTCGCACCAATTGGTCACGTTCGGCGA--ATGGTGCTTAAATTCAATTCACGGG-- 96-GTG
Azoarcus sp. T	CCGC-ACTA-GGGACAACCCCTCTTTGCTTAAGTGTTCGCACCAATTGGTCACGTTCGGCGA--ATGGTGCTTAAATTCAATTCACGGG-- 93-GTG
Hermiinimonas sp. CN	TCGC-ACCA-AGGACACCTCCCTGGTCCTTAGGTGTTTGCACCAATTGTGAT--GTGGACGA--AAGATGCTTAAATTGATAACGCTGAC- 98-GTG
"*A. aromaticum*" EbN1	-CGCCATTAAGGGCAATCCCTCCTCTCCTTAAGTGTTCGCACCAATTGGTCGCACCGAGAGA-GCCGATGCTTAAATACACCACATCGAC- 79-GTG
A. toluclasticus MF63	-CTGCATTA-GGGGCAACCCCCCCTTCGTTAAGTGTTCGCACCAATTGGTTAGGTTCAACGG--GTGGTG-CTTAATTCAATCCACGGTC- 91-GTG
T. aromatica K172	-ATACTCCA-GGGAC-CGCCCCCTATGGTTAGGTGTTCACACCAATTGCTGC--ACGATCCT--GCACGGCCTAATTTAGTCCCATGGGC- 73-GTG
T. aromatica DNT1	--AAAAGCAAGGGACATCACCACCCT-GATGGGTGTTCGCACCGATTGAAGTTGCC-ATCGAGGCAGATG-CCTAATGGAATCTCGCGAG-139-GTG
T. aromatica T1	-ATACAT-GAGGGAAATCACCACA-CCGACTGGTGATCGCACTTATTGACGCCGCC-AGCAGGGCGGAAG-TTCAATGAACCCTTACAAG-139-GTG

-35 -10 +1

***PbbsA* promoter**

Azoarcus sp. CIB	-CGTGCGGGTGGGGGTTAC-CCTTAACAGTTAGGTGTCCGCACCAATTGTTGCGCCGCAACCAGGCGCGTTTAATTGGATCTACGTTG-28-ATG
A. toluclasticus MF63	ACCTGCAGGTGAGGGTAGCCCCTTAGGCGTTAGGTGTCCGCACCAATTGTTGCAGCGCAGCCCGGCGCGTCTAATTGAATCCACGTTG-28-ATG
T. aromatica K172	CCGGGCT--GTGGGGTTGCCCCTTAGCGCCTAGGTGTTCGGACCGATTGTTGCGTTCCGGCC-TGCGGGATTTAATGGCCCCAGGTCG-28-ATG
"*A. aromaticum*" EbN1	--TGCCCCGTCGGGGTTGCCCCCTAGCGTTTAGGTGTTCGCACCAATTGTTGCCGTGTAGGCCGGCGCGTTCTAATGAACTCACGTCG-29-ATG
Hermiinimonas sp. CN	TGAACCCGGATGGGGGCACTCCTTAGTGCCTAAGTGTTCGCACCAATTGTTGCCAATAGCTTCGACGCGCAATAATGCG-TCACTTAC-30-ATG

-35 -10 +1

FIGURE S1

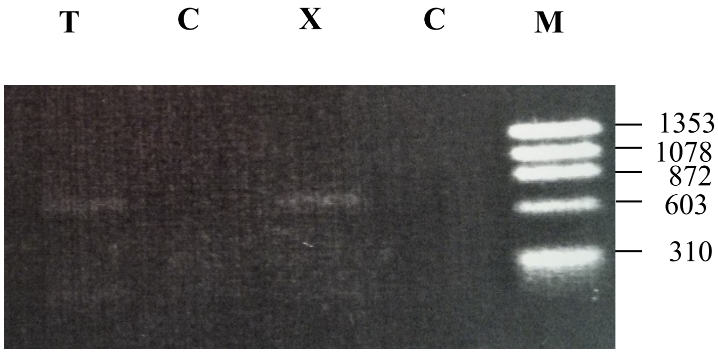

FIGURE S2

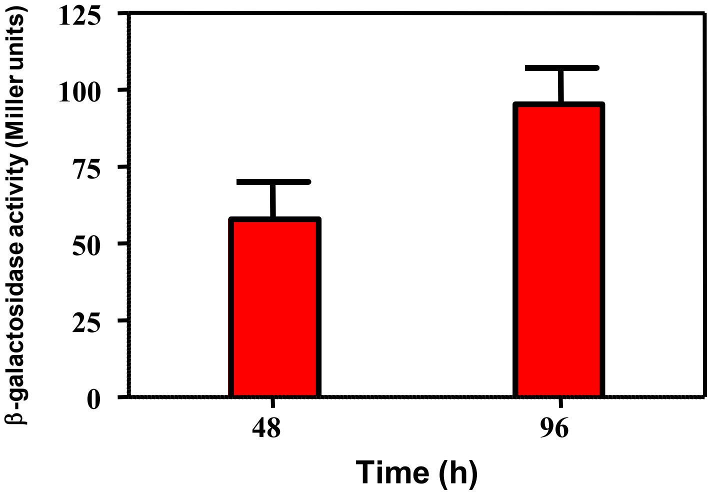
β-galactosidase activity (Miller units)
125
100
75
50
25
0
48
96
Time (h)

FIGURE S3
